# 12th WINFOCUS world congress on ultrasound in emergency and critical care

**DOI:** 10.1186/s13089-016-0046-8

**Published:** 2016-09-07

**Authors:** Yahya Acar, Onur Tezel, Necati Salman, Erdem Cevik, Margarita Algaba-Montes, Alberto Oviedo-García, Mayra Patricio-Bordomás, Mustafa Z. Mahmoud, Abdelmoneim Sulieman, Abbas Ali, Alrayah Mustafa, Ihab Abdelrahman, Mustafa Bahar, Osama Ali, H. Lester Kirchner, Gregor Prosen, Ajda Anzic, Paul Leeson, Maryam Bahreini, Fatemeh Rasooli, Houman Hosseinnejad, Gabriel Blecher, Robert Meek, Diana Egerton-Warburton, Edina Ćatić Ćuti, Stanko Belina, Tihomir Vančina, Idriz Kovačević, Nadan Rustemović, Ikwan Chang, Jin Hee Lee, Young Ho Kwak, Do Kyun Kim, Chi-Yung Cheng, Hsiu-Yung Pan, Chia-Te Kung, Ela Ćurčić, Ena Pritišanac, Ivo Planinc, Marijana Grgić Medić, Radovan Radonić, Abiola Fasina, Anthony J. Dean, Nova L. Panebianco, Patricia S. Henwood, Oliviero Fochi, Moreno Favarato, Ezio Bonanomi, Ivan Tomić, Youngrock Ha, Hongchuen Toh, Elizabeth Harmon, Wilma Chan, Cameron Baston, Gail Morrison, Frances Shofer, Angela Hua, Sharon Kim, James Tsung, Isa Gunaydin, Zeynep Kekec, Mehmet Oguzhan Ay, Jinjoo Kim, Jinhyun Kim, Gyoosung Choi, Dowon Shim, Ji-Han Lee, Jana Ambrozic, Katja Prokselj, Miha Lucovnik, Gabrijela Brzan Simenc, Asta Mačiulienė, Almantas Maleckas, Algimantas Kriščiukaitis, Vytautas Mačiulis, Andrius Macas, Sharad Mohite, Zoltan Narancsik, Hugon Možina, Sara Nikolić, Jan Hansel, Rok Petrovčič, Una Mršić, Simon Orlob, Markus Lerchbaumer, Niklas Schönegger, Reinhard Kaufmann, Chun-I Pan, Chien-Hung Wu, Sarah Pasquale, Stephanie J. Doniger, Sharon Yellin, Gerardo Chiricolo, Maja Potisek, Borut Drnovšek, Boštjan Leskovar, Kristine Robinson, Clara Kraft, Benjamin Moser, Stephen Davis, Shelley Layman, Yusef Sayeed, Joseph Minardi, Irmina Sefic Pasic, Amra Dzananovic, Anes Pasic, Sandra Vegar Zubovic, Ana Godan Hauptman, Ana Vujaklija Brajkovic, Jaksa Babel, Marina Peklic, Vedran Radonic, Luka Bielen, Peh Wee Ming, Nur hafiza Yezid, Fatahul Laham Mohammed, Zainal Abidin Huda, Wan Nasarudin Wan Ismail, W. Yus Haniff W. Isa, Hashairi Fauzi, Praveena Seeva, Mohd Zulfakar Mazlan

**Affiliations:** 1 Department of Emergency Medicine, Etimesgut Military Hospital, Ankara, Turkey; 2 Department of Emergency Medicine, Van Military Hospital, Van, Turkey; 3 Emergency Department, Hospital de Valme, Universidad de Sevilla, Seville, Spain; 4 Radiology and Medical Imaging Department, Prince Sattam bin Abdulaziz University, Al-Kharj, Saudi Arabia; 5 Critical Care Medicine, Geisinger Medical Center, Danville, USA; 6 Department of Radiology, Medical Specialization board, University of Khartoum, Khartoum, Sudan; 7 Department of Medicine, University of Khartoum, Khartoum, Sudan; 8 Department of Surgery, University of Khartoum, Khartoum, Sudan; 9 Emergency Medicine, The Academic Teaching Hospital, Khartoum, Sudan; 10 Reach Center, Geisinger Medical Center, Danville, USA; 11 Department of Emergency Medicine, University Clinical Center Maribor, Maribor, Slovenia; 12 University Medical Centre Ljubljana, Ljubljana, Slovenia; 13 Department of Cardiovascular Medicine, John Radcliffe Hospital, University of Oxford, Oxford, UK; 14 Department of Emergency, Sinai Hospital, Tehran, Iran; 15 Department of Emergency Medicine, Imam-Khomeini Hospital, Tehran, Iran; 16 Monash Emergency Research Collaborative, Monash University, Clayton, Australia; 17 Emergency Program, Monash Health, Monash Medical Centre, Clayton, Australia; 18 General Hospital Zabok and Hospital of Croatian Veterans, Zabok, Croatia; 19 Department of Veterans Affairs, Treatment Center, Miami, USA; 20 University Hospital Zagreb, Zagreb, Croatia; 21 Department of Emergency Medicine, Seoul National University Hospital, Seoul, Korea; 22 Emergency Department, Kaohsiung Chang Gung Memorial Hospital, Kaohsiung City, Taiwan; 23 Division of Intensive Care Medicine, University Hospital Centre Zagreb, Zagreb, Croatia; 24 Department of Emergency Medicine, Hospital of the University of Pennsylvania, Philadelphia, USA; 25 Department of Emergency Medicine, Brigham and Women’s Hospital, Boston, USA; 26 Pediatric intensive care, Ospedale papa giovanni XXIII, Bergamo, Italy; 27 Department of EM, Bundang Jesaeng Hospital, Seongnam, Korea; 28 Acute and Emergency Care Center, Khoo Teck Puat Hospital, Singapore, Singapore; 29 Perelman School of Medicine, University of Pennsylvania, Philadelphia, USA; 30 Department of Emergency Medicine, University of Pennsylvania, Philadelphia, USA; 31 Department of Internal Medicine, University of Pennsylvania, Philadelphia, USA; 32 Department of Emergency Medicine, Icahn School of Medicine at Mount Sinai, New York City, USA; 33 Department of Emergency Medicine, Northwell Health, New Hyde Park, USA; 34 Department of Emergency Medicine, School of Medicine, Cukurova University, Adana, Turkey; 35 Department of Emergency, Corum Education and Research Hospital, Hitit University, Corum, Turkey; 36 Gachon University Gil Medical Center, Incheon, Republic of Korea; 37 Chungbuk National University Hospital, Cheong-Ju, South Korea; 38 Department of Cardiology, University Medical Center Ljubljana, Ljubljana, Slovenia; 39 Division of Obstetrics and Gynecology, Department of Perinatology, University Medical Center Ljubljana, Ljubljana, Slovenia; 40 Department of Anaesthesiology, Lithuanian University of Health Sciences Kaunas Clinics, Kaunas, Lithuania; 41 Department of General Surgery, Lithuanian University of Health Sciences Kaunas Clinics, Kaunas, Lithuania; 42 Department of Physics, Mathematics and Biophysics, Lithuanian University of Health Sciences, Kaunas, Lithuania; 43 Department of Paediatrics, Children’s Emergency, National University Hospital, Singapore, Singapore; 44 Emergency Medical Unit, Faculty of Medicine, University Medical Centre Ljubljana, Ljubljana, Slovenia; 45 Faculty of Medicine, University of Maribor, Maribor, Slovenia; 46 Landspítali University Hospital, Reykjavík, Iceland; 47 Medical University Graz, Graz, Austria; 48 Sono4You Graz, Graz, Austria; 49 New York Methodist Hospital, Brooklyn, New York, USA; 50 Internal Medicine Department, General Hospital Trbovlje, Trbovlje, Slovenia; 51 Emergency Medicine Department, Tehran University of Medical Sciences, Tehran, Iran; 52 Department of Emergency Medicine, West Virginia University School of Medicine, Morgantown, WV USA; 53 Department of Occupational Medicine, West Virginia University School of Medicine, Morgantown, WV USA; 54 Radiology Clinic, Clinical Center University of Sarajevo, Sarajevo, Bosnia and Herzegovina; 55 Oncology Clinic, Clinical Center University of Sarajevo, Sarajevo, Bosnia and Herzegovina; 56 Dom zdravlja Centar Zagreb, Zagreb, Croatia; 57 Division of Intensive Care Medicine, KBC Zagreb, Zagreb, Croatia; 58 Singapore General Hospital, Singapore, Singapore; 59 Hospital Sultanah Bahiyah, Alor Setar, Malaysia; 60 Anaesthesiology Department, University Science of Malaysia, Kota Bharu, Malaysia

## Abstract

A1 Point-of-care ultrasound examination of cervical spine in emergency department

Yahya Acar, Onur Tezel, Necati Salman

A2 A new technique in verifying the placement of a nasogastric tube: obtaining the longitudinal view of nasogastric tube in addition to transverse view with ultrasound

Yahya Acar, Necati Salman, Onur Tezel, Erdem Cevik

A3 Pseudoaneurysm of the femoral artery after cannulation of a central venous line. Should we always use ultrasound in these procedures?

Margarita Algaba-Montes, Alberto Oviedo-García, Mayra Patricio-Bordomás

A4 Ultrasound-guided supraclavicular subclavian vein catheterization. A novel approach in emergency department

Margarita Algaba-Montes, Alberto Oviedo-García, Mayra Patricio-Bordomás

A5 Clinical ultrasound in a septic and jaundice patient in the emergency department

Margarita Algaba-Montes, Alberto Oviedo-García, Mayra Patricio-Bordomás

A6 Characterization of the eyes in preoperative cataract Saudi patients by using medical diagnostic ultrasound

Mustafa Z. Mahmoud, Abdelmoneim Sulieman

A7 High-frequency ultrasound in determining the causes of acute shoulder joint pain

Mustafa Z. Mahmoud

A8 Teaching WINFOCUS Ultrasound Life Support Basic Level 1 for Providers in resource-limited countries

Abbas Ali, Alrayah Mustafa, Ihab Abdelrahman, Mustafa Bahar, Osama Ali, H. Lester Kirchner, Gregor Prosen

A9 Changes of arterial stiffness and endothelial function during uncomplicated pregnancy

Ajda Anzic, Paul Leeson

A10 Cardiovascular haemodynamic properties before, during and after pregnancy

Ajda Anzic, Paul Leeson

A11 An old man with generalized weakness

Maryam Bahreini, Fatemeh Rasooli

A12 Ultrasonography for non-specific presentations of abdominal pain

Maryam Bahreini, Houman Hosseinnejad

A13 Introduction of a new imaging guideline for suspected renal colic in the emergency department: effect on CT Urogram utilisation

Gabriel Blecher, Robert Meek, Diana Egerton-Warburton

A14 Transabdominal ultrasound screening for pancreatic cancer in Croatian military veterans: a retrospective analysis from the first Croatian veteran’s hospital

Edina Ćatić Ćuti, Stanko Belina, Tihomir Vančina, Idriz Kovačević

A15 The challenge of AAA: unusual case of obstructive jaundice

Edina Ćatić Ćuti, Nadan Rustemović

A16 Educational effectiveness of easy-made new simulator model for ultrasound-guided procedures in pediatric patients: vascular access and foreign body management

Ikwan Chang, Jin Hee Lee, Young Ho Kwak, Do Kyun Kim

A17 Detection of uterine rupture by point-of-care ultrasound at emergency department: a case report

Chi-Yung Cheng, Hsiu-Yung Pan, Chia-Te Kung

A18 Abdominal probe in the hands of interns as a relevant diagnostic tool in revealing the cause of heart failure

Ela Ćurčić, Ena Pritišanac, Ivo Planinc, Marijana Grgić Medić, Radovan Radonić

A19 Needs assessment of the potential utility of point-of-care ultrasound within the Zanzibar health system

Abiola Fasina, Anthony J. Dean, Nova L. Panebianco, Patricia S. Henwood

A20 Ultrasonographic diagnosis of tracheal compression

Oliviero Fochi, Moreno Favarato, Ezio Bonanomi

A21 The role of ultrasound in the detection of lung infiltrates in critically ill patients: a pilot study

Marijana Grgić Medić, Ivan Tomić, Radovan Radonić

A22 The SAFER Lasso; a novel approach using point-of-care ultrasound to evaluate patients with abdominal complaints in the emergency department

Youngrock Ha, Hongchuen Toh

A23 Awareness and use of clinician-performed ultrasound among clinical clerkship faculty

Elizabeth Harmon, Wilma Chan, Cameron Baston, Gail Morrison, Frances Shofer, Nova Panebianco, Anthony J. Dean

A24 Clinical outcomes in the use of lung ultrasound for the diagnosis of pediatric pneumonias

Angela Hua, Sharon Kim, James Tsung

A25 Effectiveness of ultrasound in hypotensive patients

Isa Gunaydin, Zeynep Kekec, Mehmet Oguzhan Ay

A26 Moderate-to-severe left ventricular ejection fraction related to short-term mortality of patients with post-cardiac arrest syndrome after out-of-hospital cardiac arrest

Jinjoo Kim, Jinhyun Kim, Gyoosung Choi, Dowon Shim

A27 Usefulness of abdominal ultrasound for acute pyelonephritis diagnosis after kidney transplantation

Ji-Han Lee

A28 Lung ultrasound for assessing fluid tolerance in severe preeclampsia

Jana Ambrozic, Katja Prokselj, Miha Lucovnik

A29 Optic nerve sheath ultrasound in severe preeclampsia

Gabrijela Brzan Simenc, Jana Ambrozic, Miha Lucovnik

A30 Focused echocardiography monitoring in the postoperative period for non-cardiac patients

Asta Mačiulienė, Almantas Maleckas, Algimantas Kriščiukaitis, Vytautas Mačiulis, Andrius Macas

A31 POCUS-guided paediatric upper limb fracture reduction: algorithm, tricks, and tips

Sharad Mohite

A32 Point-of-care lung ultrasound: a good diagnostic tool for pneumonia in a septic patient

Zoltan Narancsik, Hugon Možina

A33 A case of undergraduate POCUS (r)evolution

Sara Nikolić, Jan Hansel, Rok Petrovčič, Una Mršić, Gregor Prosen

A34 The Graz Summer School for ultrasound: from first contact to bedside application: three-and-a-half-day undergraduate ultrasound training: résumé after two years of continuous development

Simon Orlob, Markus Lerchbaumer, Niklas Schönegger, Reinhard Kaufmann

A35 Usefulness of point-of-care ultrasound in the emergency room in a patient with acute abdominal pain

Alberto Oviedo-García, Margarita Algaba-Montes, Mayra Patricio-Bordomás

A36 Use of bedside ultrasound in a critically ill patient. A case report

Alberto Oviedo-García, Margarita Algaba-Montes, Mayra Patricio-Bordomás

A37 Diagnostic yield of clinical echocardiography for the emergency physician

Alberto Oviedo-García, Margarita Algaba-Montes, Mayra Patricio-Bordomás

A38 Focused cardiac ultrasound in early diagnosis of type A aortic dissection with atypical presentation

Chun-I Pan, Hsiu-Yung Pan, Chien-Hung Wu

A39 Detection of imperforated hymen by point-of-care ultrasound

Hsiu-yung Pan, Chia-Te Kung

A40 Developing a point-of-care ultrasound curriculum for pediatric nurse practitioners practicing in the pediatric emergency department

Sarah Pasquale, Stephanie J. Doniger, Sharon Yellin, Gerardo Chiricolo

A41 Use of transthoracic echocardiography in emergency setting: patient with mitral valve abscess

Maja Potisek, Borut Drnovšek, Boštjan Leskovar

A42 A young man with syncope

Fatemeh Rasooli, Maryam Bahreini

A43 Work-related repetitive use injuries in ultrasound fellows

Kristine Robinson, Clara Kraft, Benjamin Moser, Stephen Davis, Shelley Layman, Yusef Sayeed, Joseph Minardi

A44 Lung ultrasonography in the evaluation of pneumonia in children

Irmina Sefic Pasic, Amra Dzananovic, Anes Pasic, Sandra Vegar Zubovic

A45 Central venous catheter placement with the ultrasound aid: two years’ experience of the Interventional unit, Division of Intensive Care Medicine, KBC Zagreb

Ana Godan Hauptman, Marijana Grgic Medic, Ivan Tomic, Ana Vujaklija Brajkovic, Jaksa Babel, Marina Peklic, Radovan Radonic

A46 Duplicitas casui: two patients admitted due to acute liver failure

Vedran Radonic, Ivan Tomic, Luka Bielen, Marijana Grgic Medic

A47 A pilot survey on an understanding of Bedside Point-of-Care Ultrasound (POCUS) among medical doctors in internal medicine: exposure, perceptions, interest, and barriers to training

Peh Wee Ming

A48 Unusual case of defecation syncope

Nur hafiza Yezid, Fatahul Laham Mohammed

A49 A case report of massive pulmonary embolism; a multidisciplinary approach

Zainal Abidin Huda, Wan Nasarudin Wan Ismail, W.Yus Haniff W.Isa, Hashairi Fauzi, Praveena Seeva, Mohd Zulfakar Mazlan

## A1 Point-of-care ultrasound examination of cervical spine in emergency department

### Yahya Acar, Onur Tezel, Necati Salman

#### Department of Emergency medicine, Etimesgut Military Hospital, Ankara, Turkey

##### **Correspondence:** Onur Tezel, e-mail: yahyaacar@gmail.com

*Critical Ultrasound Journal* 2016, **8(Suppl 1)**:A1

**Purpose:** Cervical traumas are frequent in emergency department and X-ray, CT, and MRI are the essential imaging modalities in the diagnosis. However, especially for pregnant and morbid obese patients and children, these techniques can be challenging. We tested the success of point-of-care ultrasound in the evaluation of cervical traumas.

**Methods:** This is a case series of cervical vertebra imaging with ultrasound in emergency department. We used linear probe and placed it anterolaterally to the neck, parallel to cervical spine. Images were obtained by an ultrasound-certified emergency physician. The height of the anterior wall of vertebral body, irregularity in vertebral body, and intervertebral space were assessed.

**Results:** We presented a case series of six patients. Ultrasound images of cervical vertebral bodies and intervertebral spaces were able to obtain for all the patients. Any pathology was not observed in ultrasound imaging. This finding was compatible with cervical X-ray and CT scans and all the patients were discharged.

**Conclusions:** However, this is a case series report of minor cervical trauma, and we were able to obtain ultrasound images of cervical vertebra bodies with point-of-care ultrasound examination by an emergency physician. This technique can be important for the patients contraindicated to CT or MRI. Also, it can give additional information to X-ray and CT scans especially for soft tissues.

## A2 A new technique in verifying the placement of a nasogastric tube: obtaining the longitudinal view of nasogastric tube in addition to transverse view with ultrasound

### Yahya Acar^1^, Necati Salman^1^, Onur Tezel^1^, Erdem Cevik^2^

#### ^1^Department of Emergency medicine, Etimesgut Military Hospital, Ankara, Turkey; ^2^Department of Emergency Medicine, Van Military Hospital, Van, Turkey

##### **Correspondence:** Yahya Acar, e-mail:yahyaacar@gmail.com

*Critical Ultrasound Journal* 2016, **8(Suppl 1)**:A2

**Purpose:** Nasogastric tube (NGT) placement is performed frequently in emergency department (ED). Misplacement can cause serious complications. Many techniques were defined to verify the placement of NGT and ultrasound (US) is one of them. In this study, we reported a case and defined a new US technique as obtaining the longitudinal view in addition to the transverse view of NGT from anterolateral cervical region.

**Methods:** This is a case report of a 20-year-old man who presented to ED with vomiting after suicidal drug overdose.

**Results:** Patient’s vital signs were stable and conscious (GCS: 15). In physical examination, any abnormality could not have been detected. NGT placement was needed for gastrointestinal decontamination. The procedure was performed, and to verify the proper placement of NGT, bedside US was used. In transverse view, we had difficulty to have a clear view of the tube because of complex neck anatomy. We tried to obtain the longitudinal view of the NGT and it was better than the transverse view in visualization. In color doppler study, no flow was seen and the view of NGT was distinguished from other vascular structures. The diameter of NGT was measured and additional objective evidence was obtained. The gastric lavage procedure was accomplished and no complications were observed.

**Conclusions:** Confirmation of NGT placement is crucial and confirmation with US was reported in literature as a useful method. The procedure for obtaining the transverse view and observing the hyperechogenic “fog” was reported in literature. We recommend the use of longitudinal view and measuring the diameter of NGT in addition to the current methods.

**Informed consent** This study was conducted in accordance with the ethical standards dictated by applicable law. Informed consent was obtained from each owner for enrolment in the study and the inclusion in this article of information that could potentially lead to their identification.

## A3 Pseudoaneurysm of the femoral artery after cannulation of a central venous line. Should we always use ultrasound in these procedures?

### Margarita Algaba-Montes, Alberto Oviedo-García, Mayra Patricio-Bordomás

#### Emergency Department, Hospital de Valme, Universidad de Sevilla, Seville, Spain

##### **Correspondence:** Margarita Algaba-Montes, e-mail: margalgaba@hotmail.com

*Critical Ultrasound Journal* 2016, **8(Suppl 1)**:A3

**Purpose:** To demonstrate the utility of ultrasound when a femoral central venous line is required. Cannulation of a central venous line is required in critically ill patients in the emergency room, and it should be a basic skill for emergency physicians (EP). Its main indications include the need for rapid fluid resuscitation, central administration of drugs, and hemodynamic monitoring in critically ill patients. Like any invasive procedure, it has a risk of complications, and according to the literature, it can be very variable, ranging from 5 to 19 %, including infections, thrombosis, injury of the greater vessels, bruising, and arrhythmias, among others. There are already many studies confirming that the use of ultrasound guidance for central venous line (CVL) insertion in the emergency department reduces the rate of complications.

**Methods:** This is a case study of the diagnosis of a pseudoaneurysm of the femoral artery, which occurred after several failed attempts to insert a central venous catheter, without ultrasound, only following the classic guides for anatomical reference. The equipment used was a Sonosite M-Turbo, HLF38x linear probe 6–13 MHz.

**Results:** In this 72-year-old female, the clinical situation of hemodynamic instability, with suspected urinary sepsis, required the insertion of a CVL for hemodynamic monitoring and administration of fluids and drugs. The EP decided a femoral approach and, after several attempts, he could cannulate the vein, but within a few hours the patient developed a swelling at the puncture site. Another colleague trained in point-of-care ultrasound performed an ultrasound scan showing a large pseudoaneurysm of the femoral artery, due to accidental puncture of the artery during the previous blind procedure. After consulting with the vascular surgery service, it was decided a surgical repair was urgently required.

**Conclusions:** The current scientific literature shows that ultrasound decreases the overall incidence of complications when accessing VCL, thus making the use of this technology mandatory in many Western countries, to avoid complications, such as in the case presented. According to current recommendations of the American College of EP and other international scientific societies, the basic skills of emergency physicians must include POCUS-guided central venous access; but in Spain we are still far from achieving required numbers of emergency physicians who have been trained in the use of ultrasound in critical situations.

**Informed consent** This study was conducted in accordance with the ethical standards dictated by applicable law. Informed consent was obtained from each owner for enrolment in the study and the inclusion in this article of information that could potentially lead to their identification.

## A4 Ultrasound-guided supraclavicular subclavian vein catheterization. A novel approach in emergency department

### Margarita Algaba-Montes, Alberto Oviedo-García, Mayra Patricio-Bordomás

#### Emergency Department, Hospital de Valme, Universidad de Sevilla, Seville, Spain

##### **Correspondence:** Margarita Algaba-Montes, e-mail: margalgaba@hotmail.com

*Critical Ultrasound Journal* 2016, **8(Suppl 1)**:A4

**Purpose:** To demonstrate the utility of ultrasound in supraclavicular subclavian vein catheterization. Cannulation of a central venous line is required in critically ill patients in the emergency room, and it should be a basic skill for emergency physicians. Within the emergency care setting, the rate of placement of a central venous catheter is 1.6 per 1000 patient attendances and most emergency medicine training programmes require it as a core skill. The main indications for central venous access in the emergency department are rapid fluid resuscitation, central administration of drugs, and hemodynamic monitoring in unstable patients. Ultrasound-guided central vein cannulation has become very popular among anesthesiologists, critical care physicians, and emergency physicians in the last few years, and thus it has been advocated as the gold standard for internal jugular vein catheterization.

**Methods:** We report a technique used at our second-level hospital for cannulating subclavian vein using a supraclavicular approach under real-time ultrasound guidance. We used a Sonosite M-Turbo, HLF38x linear probe 6–13 MHz.

**Results:** On arrival, a 74-year-old female complained of severe difficulty in breathing, looked unwell, and was febrile, tachypnoeic and lethargic. Her vital signs were as follows: temperature, 38.8 °C; blood pressure, 80/38 mmHg; respiratory rate, 36 breaths per minute; room air oxygen saturation, 80 %; and heart rate, 145 beats per minute. The clinical situation of hemodynamic instability, with sepsis condition, required the insertion of a central venous line for hemodynamic monitoring and administration of fluids and drugs. In this case, we show the basic ultrasound images of the subclavian vein by supraclavicular cannulation for central venous access.

**Conclusions:** The use of ultrasound guidance for central venous cannulation is now supported by significant evidence. The cannulation of the subclavian vein by supraclavicular approach through anatomical landmarks is not commonly used for central venous access, as identification of the subclavian vein is difficult. However, it is now easily accessible by ultrasound. We would like to conclude that ultrasound-guided supraclavicular subclavian vein catheterization appears to be a safe and effective alternative for other central venous access and has become more widespread in this area, thus highlighting its potential to be considered for patients under critical conditions.

**Informed consent** This study was conducted in accordance with the ethical standards dictated by applicable law. Informed consent was obtained from each owner for enrolment in the study and the inclusion in this article of information that could potentially lead to their identification.

## A5 Clinical ultrasound in a patient with sepsis and jaundice in the emergency department

### Margarita Algaba-Montes, Alberto Oviedo-García, Mayra Patricio-Bordomás

#### Emergency Department, Hospital de Valme, Universidad de Sevilla, Seville, Spain

##### **Correspondence:** Margarita Algaba-Montes, e-mail: margalgaba@hotmail.com

*Critical Ultrasound Journal* 2016, **8(Suppl 1)**:A5

**Purpose:** To demonstrate the utility of emergency ultrasound in a patient with sepsis and jaundice. Symptoms and signs were highly suggestive of pathology of the biliary tree, but it could not be confirmed until the completion of an imaging test. Ultrasound is the preferred technique, as it provides a definitive diagnosis by showing an enlarged bile duct and a compatible image with stones inside.

**Methods:** This is a case study of a patient admitted in the emergency department with sepsis and jaundice, performing bedside ultrasound by the emergency physician, with the key tool for an early diagnosis in the emergency room. We used a Sonosite M-Turbo, with convex probe C60e/5-2 MHz.

**Results:** A 57-year-old female, with jaundice and fever for 3 days, was admitted at emergency department with a generally ill status, somnolence, tachypnea and tachycardia, and hypotension (80/50). She had abdominal pain in the right upper quadrant and showed negative Murphy’s signs, and no masses, organ enlargement, or peritonitis signs. She underwent a bedside ultrasound scan in the emergency room, which showed an anechoic tubular structure in hepatic hilum, corresponding to the dilated bile duct, of 14.3 mm, displaying inside a rounded structure hyperechoic with a posterior sonic shadow. With the diagnosis of choledocholithiasis which is complicated by acute cholangitis and biliary sepsis, the patient was treated with intensive fluid therapy, antibiotics, and drainage of the bile duct by endoscopic cholangiopancreatography; she was then discharged a few days later without any complications and with follow-up appointments for assessment of non-emergency cholecystectomy.

**Conclusions:** Point-of-care ultrasound has proven to be a useful, safe, versatile tool, and, with appropriate experience, it helps in earlier diagnosis and a comprehensive management of patients in the emergency department. By incorporating ultrasound in the emergency department, the service time can be reduced and the emergency physician can be more effective, efficient, and dynamic in the management of ‘time-dependent’ conditions, providing greater clinical patient safety. The delay in diagnosis and treatment of serious diseases could adversely affect the patient’s prognosis. It is, therefore, essential to establish and formalize training programs, with varying levels of training, which follow quality criteria, to ensure safety and efficiency of ultrasound in the hands of the emergency physician.

**Informed consent** This study was conducted in accordance with the ethical standards dictated by applicable law. Informed consent was obtained from each owner for enrolment in the study and the inclusion in this article of information that could potentially lead to their identification.

## A6 Characterization of the eyes in preoperative cataract Saudi patients by using medical diagnostic ultrasound

### Mustafa Z. Mahmoud, Abdelmoneim Sulieman

#### Radiology and Medical Imaging Department, Prince Sattam Bin Abdulaziz University, Al-Kharj, Saudi Arabia

##### **Correspondence:** Mustafa Z. Mahmoud, e-mail: m.alhassen@psau.edu.sa

*Critical Ultrasound Journal* 2016, **8(Suppl 1)**:A6

**Purpose:** This study was designed with an aim to visualize the status of the posterior portion of the eye globe of adult Saudi cataract patients with brightness mode (B-mode) ultrasound, to determine any posterior segment lesions in such preoperative cases.

**Methods:** A prospective study was performed between May 2014 and May 2015. 375 preoperative cataract (184, 49.1 %) males and (191, 50.9 %) females aged between 18 and 90 years with the mean age of 54 ± 0.5 years, were scanned at two Radiology departments. Patients were divided into nontraumatic cataract (*n* = 346; 92.3 %) and posttraumatic cataract (*n* = 29; 7.7 %) groups. B-scan ophthalmic ultrasound was performed using a Hitachi (HI Vision Avius) ultrasound machine. The statistical package for the social sciences was used to analyze the results.

**Results:** Ultrasound revealed that vitreous hemorrhage (110, 29.3 %) was the major cause of non- and posttraumatic form of cataract in preoperative adult Saudi patients. In contrast, intraocular tumors (6, 1.7 %) and posterior vitreous detachment (1, 3.5 %) were the minor causes of non- and posttraumatic cataract, respectively. Preoperative adult Saudi females are more subjective to the causes of nontraumatic cataract rather than the males in the same age, while males are able to develop posttraumatic cataract due to the abundance of etiologies in comparison to females.

**Conclusions:** Ocular ultrasound examination in preoperative cataract patients is a useful part of ophthalmic examination for detection, evaluation, and follow-up of posterior segment pathologies that may influence the surgical strategy and the postoperative visual prognosis.

## A7 High-frequency ultrasound in determining the causes of acute shoulder joint pain

### Mustafa Z. Mahmoud

#### Radiology and Medical Imaging Department, Prince Sattam bin Abdulaziz University, Al-Kharj, Saudi Arabia

##### **Correspondence:** Mustafa Z. Mahmoud, e-mail: m.alhassen@psau.edu.sa

*Critical Ultrasound Journal* 2016, **8(Suppl 1)**:A7

**Purpose:** The goal of this research was to prospectively evaluate ultrasound findings in patients with acute shoulder joint pain and also identify possible predictors of shoulder pain.

**Methods:** A sum of 65 (mean age 28 ± 1.2 years) consequential patients were recruited over a period of 6 months between July 2015 and January 2016 in this prospective study. The data collected included patient’s age, medical history, and clinical symptoms. Shoulder ultrasound was performed with a linear array transducer (10–15 MHz) connected to HI vision Avius ultrasound unit, Hitachi. MRI for the shoulder joint was performed in all cases to confirm the ultrasound results, using 1.5-T MRI system (Magnetom Espree), Siemens. Statistical analysis was performed using the standard Statistical Package for the Social Sciences transformation 20 for Windows, Microsoft.

**Results:** Ultrasound manages to determine the causes of acute shoulder joint pain in 98 % of the patients. Ultrasound in the diagnosis of the causes of shoulder joint pain showed a sensitivity of 100 % and an accuracy of 96–100 %.

**Conclusions:** Ultrasound presents high sensitivity and accuracy in diagnosing a wide spectrum of shoulder joint lesions, with a diagnostic performance near to that of MRI.

## A8 Teaching WINFOCUS Ultrasound Life Support Basic Level 1 for Providers in resource-limited countries

### Abbas Ali^1^, Alrayah Mustafa^2^, Ihab Abdelrahman^3^, Mustafa Bahar^4^, Osama Ali^5^, H. Lester Kirchner^6^, Gregor Prosen^7^

#### ^1^Critical Care Medicine, Geisinger Medical Center, Danville, USA; ^2^Department of Radiology, Medical Specialization board, University of Khartoum, Khartoum, Sudan; ^3^Department of Medicine, University of Khartoum, Khartoum, Sudan; ^4^Department of Surgery, University of Khartoum, Khartoum, Sudan; ^5^Emergency Medicine, The Academic Teaching Hospital, Khartoum, Sudan; ^6^Reach Center, Geisinger Medical Center, Danville, USA; ^7^Emergency Medicine, Maribor, Slovenia

##### **Correspondence:** Gregor Prosen, e-mail: gregorprosen@gmail.com

*Critical Ultrasound Journal* 2016, **8(Suppl 1)**:A8

**Purpose:** Our objectives are to improve the US technical and cognitive skills of physicians by conducting a WINFOCUS course in Sudan and to bring the WINFOCUS resources at the hands of physicians to improve patients’ care despite limited resources.

**Methods:** A 2-day, 16-h course of Ultrasound Life Support Basic Level 1 for Provider (USLS BL 1P) was conducted at Soba University Hospital in Khartoum/Sudan under the supervision of international and local faculty. The course utilized the ABCDE format and head-to-toe assessment with didactic lectures and hands-on training on live models according to WINFOCUS standards. The topics covered were US physics and machine handling, airway, breathing, circulation (echocardiography, FATE, AAA, DVT, and FAST), disability of the optic nerve and beyond, gall bladder and renal US, procedures by point-of-care ultrasound. We used a blue phantom model at the vascular access station in addition to a live model. The ratio of students, tutor, machine, and model was 4/1/1/1 according to WINFOCUS recommendations. To measure the improvement in the technical and cognitive skills, pre- and posttests were performed. The questions covered the material presented in the lectures and skill stations like US Physics and Knobology, image optimization, artifacts causes, and recognition.

**Results:** A total of 25 physicians were enrolled. The spectrum of specialties included critical care medicine, internal medicine, emergency medicine, pediatrics, and surgery. 18 candidates completed pre- and posttests. The mean of the pretest scores was 13.1 (SD = 5.9); the mean of the posttest scores was 19.9 (SD = 3.8); the mean of the differences between the post- and pretest scores was 7.1 (SD = 4.8, 95 % CI 4.7, 9.5) *p* < 0.001. There was a significant improvement in scores. The paired *t* test was used to compare the pre/posttest scores. Our null hypothesis was that no significant improvement in test scores would occur as a result of participation in the 2-day course.

**Conclusions:** The 2-day USLS BL-1 results in an improvement in the cognitive and technical skills of providers. We believe that true learning occurs and the improved posttest scores are not due to simple memorization. The course was well received and raised the awareness about the role of ultrasound in medical training and patients’ care in resource-limited countries. More involvement of WINFOCUS to monitor competence and certification is needed.

## A9 Changes of arterial stiffness and endothelial function during uncomplicated pregnancy

### Ajda Anzic^1^, Paul Leeson^2^

#### ^1^University Medical Centre Ljubljana, Ljubljana, Slovenia; ^2^Department of Cardiovascular Medicine, John Radcliffe Hospital, University of Oxford, Oxford, UK

##### **Correspondence:** Ajda Anzic, e-mail: ajda.anzic@gmail.com

*Critical Ultrasound Journal* 2016, **8(Suppl 1)**:A9

**Purpose:** There is clear evidence that the endothelium with its NO production has a crucial role in the maintenance of normal vascular function and that endothelial dysfunction increases arterial stiffness. Arterial stiffness is also affected by female sex steroid hormones which increase considerably in pregnancy, thus it can influence vascular properties during pregnancy. The aim of our research was to look at the changes and correlations between PWV, AIx, and endothelial function before, during, and after uncomplicated pregnancy.

**Methods:** In this longitudinal study, we quantified PWV, AIx, and endothelial function in 32 nulliparous females firstly before pregnancy, then 11–13 weeks’ gestation, 28 weeks’ gestation, and 3 months postpartum. PWV and AIx were measured with Viocorder, and endothelial function was determined with EndoCheck which records pulse volume in the brachial artery at the baseline (V1) and during reactive hyperaemia (V2).

**Results:** AIx decreased from the baseline throughout pregnancy, but it rose from baseline to significantly higher values at postpartum (*p* = 0.000). V1 progressively increased through pregnancy, but it returned to the baseline values at postpartum (*p* = 0.025). In contrast, V2 decreased from the baseline to the significantly lower values at postpartum (*p* = 0.004). V2/V1 (*p* = 0.028) and SQRT V2/V1 (*p* = 0.029) decreased throughout the pregnancy, but raised to the baseline at postpartum. The average time at which the flow-mediated dilatation was maximum was longer during pregnancy, but it returned to the baseline at postpartum (*p* = 0.038). PWV rose during pregnancy; however, its changes were not significant (*p* = 0.606). The only correlations observed were negative correlation between AIx and V1 (*r* = −0.586; *p* = 0.003) and V2 (*r* = −0.590; *p* = 0.003) at the baseline and another after delivery when AIx was positively correlated with V2/V1 (*p* = 0.027; *r* = 0.442).

**Conclusions:** During pregnancy, there were an increase in PWV and AIx and a decline in the endothelial function (lower V2/V1 and postponed maximal flow-mediated dilatation). Postpartum PWV and AIx remained significantly higher; however, V2/V1 returned to the baseline values. To summarize, normal pregnancy is associated with a significant cardiovascular adaptation indicated by alteration in endothelial function, AIx, and PWV; however, not all the values returned to the baseline after delivery.

## A10 Cardiovascular haemodynamic properties before, during and after pregnancy

### Ajda Anzic^1^, Paul Leeson^2^

#### ^1^University Medical Centre Ljubljana, Ljubljana, Slovenia; ^2^Department of Cardiovascular Medicine, John Radcliffe Hospital, University of Oxford, Oxford, UK

##### **Correspondence:** Ajda Anzic, e-mail: ajda.anzic@gmail.com

*Critical Ultrasound Journal* 2016, **8(Suppl 1)**:A10

**Purpose:** Normal pregnancy is associated with profound cardiovascular adaptation which is necessary for normal foetal growth and development. However, relatively little information is available on the changes of central hemodynamic, pulse wave velocity, augmentation index and other arterial parameters during pregnancy and post-delivery. The aim of the study was to assess the influence of pregnancy and delivery on various cardiovascular hemodynamic parameters in nulliparous women and to find if postpartum measurements significantly differ from baseline values.

**Methods:** This longitudinal study is in progress in the John Radcliffe Hospital, Oxford, UK. By April 2016, 216 non-pregnant nulliparous women (age interval 21–42 years, mean age 31.89 ± 4.87 years) have been recruited. Of these women, 65 have become pregnant during the follow-up, of whom 32 have completed pregnancy. All participants had measures of BMI, blood pressure (brachial and central), heart rate, stroke volume, cardiac output, augmentation pressure, augmentation index, total peripheral resistance and pulse wave velocity before pregnancy, 11–13 weeks’ gestation, 28 weeks’ gestation and 3 months postpartum.

**Results:** There was a statistically significant difference between all visits in BMI (*p* = 0.001), brachial, peripheral and central SBP (*p* = 0.004; *p* = 0.001; *p* = 0.000) and DBP (*p* = 0.000; *p* = 0.004; *p* = 0.004), peripheral and central PP (*p* = 0.005; *p* = 0.006), HR (*p* = 0.006), SV (*p* = 0.009), AP (*p* = 0.029), AI (*p* = 0.000) and total peripheral resistance (*p* = 0.022). There were no statistically significant differences in CO (*p* = 0.328) and PWV (*p* = 0.606). Comparison of baseline and postpartum measurements showed a statistically significant increase of BMI (*p* = 0.001), central SBP (*p* = 0.004), peripheral and central DBP (*p* = 0.012 and *p* = 0.006), peripheral MAP (*p* = 0.011), HR (*p* = 0.037), AP (*p* = 0.000), AI (*p* = 0.000) and PWV (*p* = 0.020).

**Conclusions:** This study is one of the first studies with longitudinal measures of cardiovascular haemodynamics in nulliparous women from before conception to postpartum. Our data show that it is feasible to perform serial measurements in this setting and that several key cardiovascular parameters change significantly in normal pregnancy between pre-pregnancy and postpartum.

## A11 An old man with generalized weakness

### Maryam Bahreini, Fatemeh Rasooli

#### ^1^Department of Emergency, Sinai Hospital, Tehran, Iran

##### **Correspondence:** Maryam Bahreini, e-mail: bahreinimaryam@gmail.com

*Critical Ultrasound Journal* 2016, **8(Suppl 1)**:A11

**Purpose:** Weakness is a nonspecific complaint, resulting from various leading emergent to nonurgent causes, so that its approach and management depends mainly on current and underlying patient status. In this case, an odd presentation of massive pericardial effusion with weakness and trivial cardiac symptoms is presented, which was diagnosed mainly by bedside ultrasonography.

**Methods:** A 68-year-old man was transferred to emergency department because of generalized symmetrical weakness. He did not have any complaints of chest pain, dyspnea, or fever. He became bedridden because of exertional fatigue in the past month. No other respiratory, gastrointestinal, or neurologic complaint was noted, but constitutional symptoms were present. Past history was negative and he declared heavy smoking habit. His vital signs were a blood pressure of 100/60 mmHg with pulsus paradoxus of 40 mmHg, a heart rate 100 of beats per minute, a respiratory rate of 32 breaths per minute, and the room air oxygen saturation of 92 %. On examination, he was an ill, cachectic man with no remarkable physical finding: no edema was present, jugular vein distention was noted, no ophthalmopathy, thyromegaly, or lymphadenopathy was found, lungs were clear, heart sounds were muffled, and he did not have any focal neurologic deficit.

**Results:** Lab data showed the following nonspecific findings: he had mild normochromic normocytic anemia, an elevated sedimentation rate, and normal cardiac enzymes and D-Dimer. Electrocardiography findings were sinus tachycardia, relative low-voltage complexes without obvious electrical alternans, or ST-T change. Urine toxicology was negative. Chest X-ray exhibited cardiomegaly without further obvious pathology. Bedside sonography was performed, which revealed a large pericardial effusion without right atrial or ventricular collapse. Being relatively stable, he was admitted to surgery ward for further diagnostic management and therapeutic drainage. Finally, the leading cause of massive pericardial effusion was found to be mediastinal lymphoma.

**Conclusions:** Nonspecific complaints are relatively common among emergency patients and result in higher morbidity and mortality rates. This patient showed subtle symptoms and vague presentation of a large pericardial effusion that was revealed by point-of-care ultrasonography. Therefore, the most helpful diagnostic modality resulting in rapid determination of patient disposition was emergency ultrasound guidance in accordance with clinical manifestations.

**Informed consent** This study was conducted in accordance with the ethical standards dictated by applicable law. Informed consent was obtained from each owner for enrolment in the study and the inclusion in this article of information that could potentially lead to their identification.

## A12 Ultrasonography for non-specific presentations of abdominal pain

### Maryam Bahreini^1^, Houman Hosseinnejad^2^

#### ^1^Department of Emergency, Sinai Hospital, Tehran, Iran; ^2^Department of Emergency Medicine, Imam-Khomeini Hospital, Tehran, Iran

##### **Correspondence:** Maryam Bahreini, e-mail: bahreinimaryam@gmail.com

*Critical Ultrasound Journal* 2016, **8(Suppl 1)**:A12

**Purpose:** A wide range of challenging differential diagnoses exist for acute abdominal pain in the elderly patients presenting to an overcrowded emergency department. These settings prompt rapid bedside diagnostic modalities to rule out emergent causes and save time for further assessment of nonurgent measures. Here, we present a nonspecific presentation of abdominal pain in which differential diagnoses were reviewed by bedside sonography until achievement of final disposition.

**Methods:** An 80-year-old woman was presented to our emergency department with right flank pain that was radiated to epigastric region and back from 2 days. The abdominal pain was severe and constant, not relieved with proton pump inhibitors or nitrates and did not depend on activity or feeding. Nausea and vomiting were persistent till the day before but there was no loss of appetite. Her bowel movement and passing gas were normal. No chest pain, dyspnea, or dyspepsia was noted. Vital signs were within normal limits. Past history was positive for hypertension and balloon coronary angioplasty at the age of 5 months. On physical exam, a mild epigastric tenderness without rebound tenderness or guarding was noted and no palpable pulsatile mass was present.

**Results:** Electrocardiography (ECG) had nonspecific ST-T changes compatible with previous ECGs. Lab data were within normal limits including lipase, cardiac troponin, and liver function tests. Chest X-ray showed no specific abnormal finding. On bedside sonography, no pericardial effusion was noted, aorta size was normal without evidence of mural thrombosis or false lumen, no hydronephrosis or stone was remarked in the right kidney, and gallbladder scan showed increased wall thickness and multiple tiny gallstones with posterior shadows without pericholecystic fluid. Ultrasound imaging of pancreas, biliary tree, and common bile duct was normal. Finally, she was referred to a surgeon for further management.

**Conclusions:** Acute upper abdominal pain in the elderly has several differential diagnoses ranging from critical to nonurgent conditions. The decision to define final management and disposition in an overcrowded emergency department must be done case by case and bedside ultrasonography is a useful tool for a stepwise emergency approach.

**Informed consent** This study was conducted in accordance with the ethical standards dictated by applicable law. Informed consent was obtained from each owner for enrolment in the study and the inclusion in this article of information that could potentially lead to their identification.

## A13 Introduction of a new imaging guideline for suspected renal colic in the emergency department: effect on CT Urogram utilisation

### Gabriel Blecher^1,2^, Robert Meek^1,2^, Diana Egerton-Warburton^1,2^

#### ^1^Monash Emergency Research Collaborative, Monash University, Clayton, Australia; ^2^Emergency Program, Monash Health, Monash Medical Centre, Clayton, Australia

##### **Correspondence:** Gabriel Blecher, e-mail: gabriel.blecher@monashhealth.org

*Critical Ultrasound Journal* 2016, **8(Suppl 1)**:A13

**Purpose:** To compare computed tomography urography (CTU) use between the Monash Clayton Emergency Department (ED), following the introduction of a revised guideline for suspected renal colic, aimed at restricting CTU use to those with higher likelihood of needing a urological intervention, and the Monash Dandenong ED, where the use of the current guideline was continued.

**Methods:** A quasi-experimental cohort study was conducted on a consecutive series of eligible patients presenting to two Monash Health EDs from November 2015 until February 2016. The study site was Monash Clayton and the control site was Monash Dandenong. All patients who were aged 18 or over who presented to the emergency department with renal colic clinically suspected by the treating clinician were eligible for recruitment. A new imaging protocol was developed subsequent to prior work in our institution, which incorporated point-of-care ultrasound to detect hydronephrosis and AAA. CTU was performed for red flag presence or failure to achieve adequate analgesia. The protocol was promoted in medical and nursing education sessions solely at the Monash site prior to study commencement. Routine care was provided to all patients with suspected renal colic at the Monash Dandenong site. The primary outcome measure was the difference in the proportion of patients with suspected renal colic undergoing CTU between the two sites. Secondary outcomes include the following:

1. proportion of patients having CTU who have ureteric stones detected,

2. proportion of patients having CTU who have a urological intervention,

3. proportion of patients who return to ED,

4. time to urological intervention,

5. average radiation exposure per patient,

6. admission rates, and

7. ED length of stay (LOS).

**Results:** A total of 325 encounters were recorded, 149 (45.9 %) at Monash and 176 (54.2 %) at Dandenong. 71.1 % were male and the mean age was 48 years. CTU was performed in 80 (53.7 %) Monash patients and in 132 (75.0 %) Dandenong patients (*p* < 0.001). The CTU was positive for calculus in 61 (76.3 %) Monash patients and in 83 (62.9 %) Dandenong patients (p = 0.043). The median radiation dose was 2.6 mSv in Monash patients and 4.0 mSv in Dandenong patients (*p* < 0.001). Urological intervention occurred in 10 (12.4 %) Monash and 13 (11.4 %) Dandenong patients (*p* = 0.84).

**Conclusions:** A new ultrasound-first imaging protocol in renal colic successfully reduces CTU use, with an attendant drop in radiation dose and an increased proportion of CTU showing calculi.

## A14 Transabdominal ultrasound screening for pancreatic cancer in Croatian military veterans: a retrospective analysis from the first Croatian veteran’s hospital

### Edina Ćatić Ćuti^1^, Stanko Belina^1^, Tihomir Vančina^1^, Idriz Kovačević^2^

#### ^1^General Hospital Zabok and Hospital of Croatian Veterans, Zabok, Croatia; ^2^Department of Veterans Affairs, Treatment Center, Miami, USA

##### **Correspondence:** Edina Ćatić Ćuti, e-mail: edina3110@gmail.com

*Critical Ultrasound Journal* 2016, **8(Suppl 1)**:A14

**Background:** General hospital Zabok was recently designated as a first military veteran’s hospital in Croatia. Approximately 10 000 military veterans received a broad array of medical services since January 2015.

**Purpose:** We performed a retrospective analysis of the use of transabdominal ultrasound in the detection of smoking-related abdominal malignancies in Croatian military veterans undergoing annual physical examination. According to the literature, military veteran’s population has higher incidence of tobacco use. Also, smoking is a known risk factor for pancreatic cancer that usually goes undetected until advanced stage and is the fourth most common cause of cancer mortality. The goal of the study is to assess whether the inclusion of transabdominal ultrasound into annual physical examination improves the detection of pancreatic cancer in veterans with positive smoking history.

**Methods:** 168 transabdominal ultrasound examinations were completed in the period from January 2015 to December 2015. All transabdominal US were completed as a part of veteran’s annual physical examination. Demographic characteristics and smoking status of patients were assessed.

**Results:** In this sample of 168 cases, we found two suspected pancreatic tumors that required further gastroenterology evaluation and in one case pancreatic cancer was confirmed.

**Conclusions:** The analysis did not find higher incidence of pancreatic cancer in the small sample of 168 patients. Future research should address the feasibility of inclusion of transabdominal US into annual physical examination of veterans with positive tobacco history and otherwise negative gastrointestinal history, physical exam, and laboratory values.

## A15 The challenge of AAA: unusual case of obstructive jaundice

### Edina Ćatić Ćuti^1^, Nadan Rustemović^2^

#### ^1^General Hospital Zabok and Hospital of Croatian Veterans, Zabok, Croatia; ^2^University Hospital Zagreb, Zagreb, Croatia

##### **Correspondence:** Edina Ćatić Ćuti, e-mail: edina3110@gmail.com

*Critical Ultrasound Journal* 2016, **8(Suppl 1)**:A15

**Purpose:** Abdominal aortic aneurysm (AAA) is an important clinical entity with serious complications that can easily be misdiagnosed due to variable clinical presentations, from being asymptomatic to severe abdominal or back pain. The most serious complication leading to the fatal outcome is AAA rupture. There is an interesting quote by JJ Duphie: ‘A significant number of lives might be saved if clinicians were more aware of this possibility’. A rare complication is obstructive jaundice due to external compression; actually, we found a single publication on this issue, reported by Reiβ and colleagues. Our presentation focuses on the importance of transabdominal ultrasound in the early diagnosis of obstructive jaundice as a rare complication of AAA.

**Methods:** We present a case of a 66-year-old male patient, who is a heavy smoker, with new-onset painless jaundice followed by cholestatic pattern in laboratory tests. Transabdominal ultrasound verified AAA, 9 cm in diameter, with thrombotic masses and a visible dilatation of intra- and extrahepatic ducts, what was confirmed also by MSCT. No malignancy was confirmed by further diagnostic procedures, including MRI. Our gastroenterology and surgery team decided first to preform AAA repair and then to continue with diagnostic evaluation of other possible causes of obstructive jaundice.

**Results:** After the AAA repair, a significant decrease in total bilirubin within the reference range and a gradual decrease of ALP and GGT levels were observed, which led us to the conclusion that AAA may have contributed to the clinical presentation of obstructive jaundice by means of external compression. Control ultrasound assessment showed biliary duct dilatation, probably due to known stenosis in the middle part, followed by again a laboratory increase of ALP and GGT. ERCP was recommended and successfully preformed with insertion of plastic biliary stent. Brush cytology did not confirm malignant etiology. The patient achieved an overall good recovery.

**Conclusions:** In view of the above, transabdominal ultrasound is shown to be an important low-cost and noninvasive reproducible tool in diagnostic algorithm for the patients with obstructive jaundice.

**Informed consent** This study was conducted in accordance with the ethical standards dictated by applicable law. Informed consent was obtained from each owner for enrolment in the study and the inclusion in this article of information that could potentially lead to their identification.

## A16 Educational effectiveness of easy-made new simulator model for ultrasound-guided procedures in pediatric patients: vascular access and foreign body management

### Ikwan Chang, Jin Hee Lee, Young Ho Kwak, Do Kyun Kim

#### ^1^Department of Emergency Medicine, Seoul National University Hospital, Seoul, Korea

##### **Correspondence:** Ikwan Chang, e-mail: taketime97@gmail.com

*Critical Ultrasound Journal* 2016, **8(Suppl 1)**:A16

**Purpose:** This study was aimed to introduce an easy-made chicken breast model for ultrasound (US)-guided vascular access, foreign body (FB) detection, and hydro-dissection in pediatric patients, and to evaluate the effectiveness of education using this phantom model.

**Methods:** The authors made the tissue phantom model using a chicken breast by a very simple method. We used rubber tourniquet for vascular access, and a tongue blade and steel clip for FB detection and hydro-dissection. We provided the education of US-guided vascular access [following the tip (FTT) method], FB detection, and hydro-dissection using this model to novice physicians to perform US-guided procedure in pediatric patients. And, we distributed the questionnaire about their thinking of own knowledge and confidence in this procedure before and after education, and their thinking of the reality and usefulness of this model on a 10-point Likert scale.

**Results:** A total of 16 emergency residents participated in this education. The median difference scores in knowledge of US-guided FTT method, FB detection, and hydro-dissection between pre- and post-education were 5 (IQR 3.5, 7) (*p* < 0.001), 5 (IQR 3, 8) (*p* < 0.001), and 5.5 (IQR 4.5, 8), respectively, and the median difference scores in confidence were 5 (IQR 4, 7) (*p* < 0.001) in US-guided FTT, 5.5 (IQR 3.5, 7) (*p* < 0.001) in US-guided FB detection, and 5 (IQR 4, 7) (*p* < 0.001) in US-guided hydro-dissection. The median Likert scores for the question regarding the reality and usefulness of this model were 8.5 (IQR 7.5, 9) and 10 (IQR 8, 10), respectively.

**Conclusions:** We made the model for US-guided procedures by simple and easy methods. Using this model could provide the novice physicians with a realistic training for US-guided procedure in pediatric patients.

## A17 Detection of uterine rupture by point-of-care ultrasound at emergency department: a case report

### Chi-Yung Cheng^1^, Hsiu-Yung Pan^2^, Chia-Te Kung^3^

#### ^1^Emergency Department, Kaohsiung Chang Gung Memorial Hospital, Kaohsiung City, Taiwan; ^2^Emergency Department, Kaohsiung Chang Gung Memorial Hospital, Kaohsiung City, Taiwan; ^3^Kaohsiung Chang Gung Memorial Hospital, Kaohsiung City, Taiwan

##### **Correspondence:** Chi-Yung Cheng, e-mail: qzsecawsxd@cgmh.org.tw

*Critical Ultrasound Journal* 2016, **8(Suppl 1)**:A17

**Purpose:** Uterine rupture is a life-threatening pregnancy complication for both mother and fetus. The clinical presentations of uterine rupture vary greatly, which makes the correct diagnosis difficult. Detection of hemoperitoneum using point-of-care ultrasound (POC-US) may be life saving in this obstetric emergency.

**Methods:** This is a case report of a 39-year-old woman, G2P1 (via Cesarean delivery), referred from primary hospital, suspecting of intrauterine fetal demise at 26 weeks’ gestation. She underwent cesarean section (C/S) 12 years ago due to fetal breech presentation and received laparoscopic myomectomy 2 months ago before conception. She suffered from low abdominal pain for 1 day and was referred to our emergency department (ED) from primary hospital where intrauterine fetal demise was suspected. Nonviable pregnancy was confirmed by consulted obstetrician and admission for termination was suggested. Worsened abdominal pain with peritonitis developed later, and point-of-care ultrasound (POC-US) was performed by ED physician. Fluid in the cul-de-sac and suspicious dehiscence of uterine wall over the right fundus are shown. Emergent laparotomy was performed under the impression of uterine rupture with internal bleeding, and rupture over the right fundal area with a dead male baby was found during operation. She was discharged days later and an improvement was documented in the medical record at Outpatient Department.

**Results:** Uterine rupture during pregnancy is a obstetric emergency with high maternal and fetal mortality. The main risk factors for uterine rupture are previous uterine rupture, previous fundal or vertical hysterotomy, induction, and labor. If the fetal and maternal conditions are unstable and uterine rupture is suspected, POC-US may be used for real-time images and dynamic changes. Our patient underwent C/S 12 years ago and received laparoscopic myomectomy to remove a right fundal myoma 2 months ago before conception. The previous uterine surgeries made her to have a higher risk of developing uterine rupture. POC-US allows ED physicians to rapidly identify hemoperitoneum and expedite the operative management in this patient.

**Conclusions:** POC-US can provide real-time images and dynamic changes for pregnant woman in ED, and may be used as the first-line tool to screen obstetric emergency.

**Informed consent** This study was conducted in accordance with the ethical standards dictated by applicable law. Informed consent was obtained from each owner for enrolment in the study and the inclusion in this article of information that could potentially lead to their identification.

## A18 Abdominal probe in the hands of interns as a relevant diagnostic tool in revealing the cause of heart failure

### Ela Ćurčić, Ena Pritišanac, Ivo Planinc, Marijana Grgić Medić, Radovan Radonić

#### ^1^Division of Intensive Care Medicine, University Hospital Centre Zagreb, Zagreb, Croatia

##### **Correspondence:** Ela Ćurčić, e-mail: elacurcic@gmail.com

*Critical Ultrasound Journal* 2016, **8(Suppl 1)**:A18

**Purpose:** To demonstrate the importance of ultrasound training for medical students and young interns and its diagnostic value in emergency department setting.

**Methods:** A 59-year-old male patient was admitted to the intensive care unit (ICU) from the emergency department where he presented with acutely decompensated heart failure. His general condition deteriorated 10 days prior to admission, with flu-like symptoms followed by swelling of both legs. Nocturnal dyspnea was the patient’s leading symptom. He experienced no chest pain, palpitations, or syncope. Physical examination revealed dyspnea, inspiratory crackles, quiet heart sounds without murmurs, palpable liver, and pretibial pitting edema. Initial laboratory findings showed a troponin T level of 0.025 µg/L and microcytic anemia (Hb 108 g/L, MCV 79.4 fL). Four months earlier, he was hospitalized with inferolateral myocardial infarction, treated with PCI (two stents inserted in the LAD). Echocardiography showed hypocontractile inferoposterior segment of the LV, with a LVEF of 65 %. After hospital discharge, the patient did not manifest any signs of CAD until reported admission. Additionally, medical history confirmed arterial hypertension, hyperlipidemia, poorly regulated diabetes type II, colon polyps, and hemorrhoids (with consequential anemia). Parenteral diuretic therapy was initiated and the patient was admitted to the ICU where interns, who had recently attended USLS-BL1, immediately performed bedside ultrasound as a part of a routine patient checkup. They used convex abdominal probe.

**Results:** The patient demonstrated common sonographic images of volume overload (B lines, dilated and poorly collapsing inferior vena cava during inspiration). Unexpectedly, subxiphoid window revealed a bizarre round formation in the apical segment of the LV, with an echogenic mass in it. More meticulous examination performed afterwards by the attending intensivist and echocardiographer confirmed an aneurysm of the LV containing a mural thrombus. Cardiac MRI finding was complementary. The patient underwent a LV aneurysmectomy after which his condition significantly improved.

**Conclusions:** Ultrasound screening performed by relatively inexperienced young physicians detected abnormality of the heart in the patient who initially presented with typical symptoms of heart failure. This finding influenced the clinical decision-making process significantly, directing further procedures toward surgical treatment.

**Informed consent** This study was conducted in accordance with the ethical standards dictated by applicable law. Informed consent was obtained from each owner for enrolment in the study and the inclusion in this article of information that could potentially lead to their identification.

## A19 Needs assessment of the potential utility of point-of-care ultrasound within the Zanzibar health system

### Abiola Fasina^1^, Anthony J. Dean^1^, Nova L. Panebianco^1^, Patricia S. Henwood^2^

#### ^1^Department of Emergency Medicine, Hospital of the University of Pennsylvania, Philadelphia, USA; ^2^Department of Emergency Medicine, Brigham and Women’s Hospital, Boston, USA

##### **Correspondence:** Anthony J. Dean, e-mail: anthony.dean@uphs.upenn.edu; ajdean2@gmail.com

*Critical Ultrasound Journal* 2016, **8(Suppl 1)**:A19

**Purpose:** The availability of diagnostic imaging in Zanzibar is currently limited. Point-of-care ultrasound (POCUS) is accurate, safe, portable, and relatively inexpensive compared to other imaging modalities. These features make it ideal for resource-limited settings. The Zanzibar Ministry of Health has identified access to ultrasound (US) as a health system priority. This study assessed health care providers’ (HCPs) current training and use of US in Zanzibar, as well as barriers to the implementation of POCUS.

**Methods:** A quantitative survey was administered on site at the 8 public hospitals in Zanzibar to a convenience sample of HCPs in July 2015. A previously published US needs assessment survey tool was modified for the current study. HCPs also completed focused personal interviews after survey completion to attain further qualitative data.

**Results:** Forty individuals completed the survey for a response rate of 97.5 %. Survey data revealed that prior experience with POCUS was poor with 92.3 % reporting no prior ultrasound training or experience. The majority of those surveyed (70 %) indicated a ‘high’ interest in learning ultrasound. Of those reporting interest in POCUS applications (*n* = 35), obstetrics was the most popular (71.4 %). The lack of ultrasound machines and educators were identified as the greatest barriers to US utilization (39.7 and 27.6 %, respectively). Obstetrics and evaluation of peritonitis are currently the most common indications for US.

**Conclusions:** HCPs in Zanzibar have limited access to US and express a high level of interest in learning the skill. A shortage of machines and educators are the main barriers to more widespread use. Obstetrics is the application in which US is currently most used and which the greatest number are interested to learn.

## A20 Ultrasonographic diagnosis of tracheal compression

### Oliviero Fochi, Moreno Favarato, Ezio Bonanomi

#### Pediatric intensive care, Ospedale Papa Giovanni XXIII, Bergamo, Italy

##### **Correspondence:** Oliviero Fochi, e-mail: ofochi@hpg23.it

*Critical Ultrasound Journal* 2016, **8(Suppl 1)**:A20

**Purpose:** To highlight an application of ultrasonography in the detection of pediatric ingested foreign bodies.

**Methods:** This is a brief clinical case report.

**Results:** A 3-year-old child, with a history of surgery for esophageal atresia, suffered an episode of sudden respiratory distress, severe cyanosis, and loss of consciousness at home while eating potatoes. He was resuscitated by the Emergency Medical Service and transported to our hospital, where he was intubated, stabilized, and transferred to our Intensive Care Unit. Chest X-ray was unremarkable, while pulmonary ultrasound showed a right upper lobe atelectasis. Bronchoscopy revealed a compression of the lower two-thirds of a seemingly malacic trachea, extending to the carina and the right main bronchus. No foreign body was visible. Bedside neck ultrasound was then performed and a large food bolus with a uniform hypoechoic structure was found in the esophagus at the thoracic inlet. The potato was removed with esophagogastroduodenoscopy and the esophagus cleared. The patient had a complete recovery and was discharged on day 2.

**Conclusions:** Neck ultrasonography can allow direct visualization of an ingested foreign body within the esophagus. In this paper, we show clear images of an esophagus dilated by a potato and subsequently cleared by esophagogastroscopy.

Bibliography

Fochi O, Favarato M, Bonanomi E (2016) Ultrasonographic diagnosis of tracheal compression. Intensive Care Med

**Informed consent** This study was conducted in accordance with the ethical standards dictated by applicable law. Informed consent was obtained from each owner for enrolment in the study and the inclusion in this article of information that could potentially lead to their identification.

## A21 The role of ultrasound in the detection of lung infiltrates in critically ill patients: a pilot study

### Marijana Grgić Medić, Ivan Tomić, Radovan Radonić

#### ^1^University Hospital Centre Zagreb, Zagreb, Croatia

##### **Correspondence:** Marijana Grgić Medić, e-mail: marijana.grgic@gmail.com

*Critical Ultrasound Journal* 2016, **8(Suppl 1)**:A21

**Purpose:** The diagnosis of pulmonary infiltrates often requires CT scanning since clinical data can be unreliable, particularly in critically ill and immunosuppressed patients, and bedside chest radiography (CXR) can be misleading. The aim of this study was to evaluate the role of bedside chest ultrasound in the detection of pulmonary inflammatory infiltrates in critically ill patients admitted for acute respiratory failure, in comparison with CXR, using the chest computed tomography (CT) as the reference point.

**Methods:** During 6 months, 28 patients (14 female) aged 65 ± 14 years, admitted to the ICU for acute respiratory failure, were prospectively studied. All patients had a chest CT performed for clinical purposes. The lung ultrasound was performed at admission to the ICU or within 24 h from the CT scan by the intensivist trained at USLS-BL1, who was unaware of the CT results. The bedside CXR was performed within 1 h from the ultrasound. Ultrasound results were positive in case of (1) inhomogenous, localised B lines with local pleural abnormalities; (2) lung consolidates—tissue-like lung pattern and air bronchogram; and (3) interstitial or interstitial/alveolar pattern—bilateral confluent B lines with pleural abnormalities, indicating ARDS. Chest CT and CXR results were interpreted by a radiologist unaware of the ultrasound results.

**Results:** Pulmonary inflammatory infiltrates were detected by CT in 15 patients (15/28). Ultrasound detected pulmonary infiltrates in 14/15 patients (consolidates in 12 and bilateral B lines originating from pleural line in 2 patients). Out of the 13 patients with negative CT results, ultrasound examination was negative in 12/13 patients and was positive (unilateral basal lung consolidation) in 1 patient. Sensitivity of ultrasound in the detection of pulmonary infiltrates was 93 % and specificity was 92 %. Chest X-ray detected pulmonary infiltrates in 9/15 patients with positive CT and was negative in 12/13 patients with negative CT results. The sensitivity of CXR was 60 % and specificity was 92 %.

**Conclusions:** Bedside ultrasound appears to be a reliable tool in the evaluation of lung infiltrates in the critically ill patients, and could be superior to chest radiography. The routine use of bedside US by a trained intensivist could improve the diagnostic evaluation of pulmonary pathology, allow us to avoid the unnecessary irradiation of patients, and initiate the timely therapy.

## A22 The SAFER Lasso; a novel approach using point-of-care ultrasound to evaluate patients with abdominal complaints in the emergency department

### Youngrock Ha^1^, Hongchuen Toh^2^

#### ^1^Department of EM, Bundang Jesaeng Hospital, Seongnam, Korea; ^2^Acute and Emergency Care Center, Khoo Teck Puat Hospital, Singapore, Singapore

##### **Correspondence:** Youngrock Ha, e-mail: youngrock.ha@gmail.com

*Critical Ultrasound Journal* 2016, **8(Suppl 1)**:A22

**Purpose:** Ultrasound is an useful imaging modality to evaluate patients presenting to the emergency department (ED) with abdominal complaints. While there are attempts to conceptualize a systematic approach using point-of-care ultrasound (POCUS) in this setting, to our best knowledge there is no literature describing how it could be performed in a focused manner.

**Results:** We develop a focused (SAFER) and systematic (Lasso) framework to evaluate adult patients with abdominal complaints using POCUS. The evaluation of an organ begins with “S” or size. “A” evaluates for air that is free within the adjacent peritoneal space or trapped within the organ. Similarly, “F” assesses for free fluid (peritoneal) or trapped fluid (organ). Echogenicity (E) should be homogenous in solid organs. “R” assesses for regional lesion, either as a discrete mass, cyst, or calcification, or indiscreetly as a distortion of the normal organ contour. “Lasso,” a loop of rope that is designed to be thrown around a target (targeted diseases), directs the sequence of scan. We start at the epigastrium with the left liver lobe, stomach, inferior vena cava, and pancreas, and then the right liver lobe, gallbladder, biliary tract, and the right kidney. Next is the spleen, left kidney, abdominal aorta, small bowels, pelvic organs, appendix, and finally the large bowel.

**Discussion:** After the introduction of Focused Assessment with Sonography in Trauma, the abdominal application of POCUS has expanded beyond identification of free fluid in the peritoneal space and has potentially increased the diagnostic confidence of physicians by the bedside. Recent attempts in this field are directed at performing it in a systematic fashion (“where to look”), but did not inform sufficiently on the actual assessment of the organs (“how to look”). With our understanding of POCUS as essentially a procedural skill that yields the fruits of a focused point-of-care test, a simple and targeted approach is required. Drawing on our own clinical and teaching experience, we created the SAFER Lasso framework to assist the clinicians in performing bedside ultrasound evaluation of patients with abdominal complaints. The organs are systematically scanned (Lasso) and interrogated in the five aspects (SAFER) described above, each yielding a yes–no answer.

**Conclusions:** The SAFER Lasso is a focused and systematic evaluation of the abdominal organs and peritoneal space using POCUS.

## A23 Awareness and use of clinician-performed ultrasound among clinical clerkship faculty

### Elizabeth Harmon^1^, Wilma Chan^1,2^, Cameron Baston^1,3^, Gail Morrison^1^, Frances Shofer^1,2^, Nova Panebianco^1,2^, Anthony J. Dean^1,2^

#### ^1^Perelman School of Medicine, University of Pennsylvania, Philadelphia, USA; ^2^Department of Emergency Medicine, University of Pennsylvania, Philadelphia, USA; ^3^Department of Internal Medicine, University of Pennsylvania, Philadelphia, USA

##### **Correspondence:** Anthony J. Dean, e-mail: anthony.dean@uphs.upenn.edu; ajdean2@gmail.com

*Critical Ultrasound Journal* 2016, **8(Suppl 1)**:A23

**Purpose:** Clinician-performed ultrasound (CPU) is an increasingly widely used tool in many specialties. Capacity for CPU education during medical school clerkships is unknown. Clerkship students may lack clinical CPU education because faculty lack formal training. This study seeks to assess current practice patterns and knowledge of CPU indications among clinical clerkship faculty (CCF).

**Methods:** This was a web-based, cross-sectional survey targeting CCF in educational leadership positions from five specialties [Internal Medicine, Surgery, Family Medicine, Emergency Medicine (EM), and Pediatric EM] in a single medical school. The survey examined CCFs’ demographics, comfort using and interpreting CPU, frequency of CPU use, and knowledge of 12 widely accepted CPU indications and 3 “false indications” (diseases not known to benefit from CPU). Knowledge of CPU indications was based on a 4-point Likert scale from 0 (“Never use CPU”) to 3 (“Always use CPU”). The 22-item survey was developed by content experts and was pilot tested prior to distribution. Results were analyzed with standard statistical methods.

**Results:** Forty-six percent (88/192) of invited CCF responded. Overall, 68 and 72 % of respondents felt uncomfortable or only somewhat comfortable performing and interpreting CPU, respectively. Comfort performing CPU varied by medical specialty, with a significantly higher comfort level among EM and Pediatric EM physicians (*p* = 0.0002). Awareness of when to use CPU for true indications ranged from a high of 2.14 (often to always) for cardiac tamponade and a low of 0.25 (never to sometimes) for elevated intracranial pressure. The respondents appropriately avoided CPU for “false indications.” Both comfort level and knowledge of CPU indications decreased with increasing years since residency. More recent graduates (0–15 years since residency) were significantly more comfortable using CPU than the more senior respondents (15+ years since residency) (*p* = 0.0029).

**Conclusions:** Half of CCF in educational leadership positions are uncomfortable performing and interpreting CPU. CPU comfort and awareness decreases with increasing years since residency and is higher in EM and Pediatric EM physicians. Many time-sensitive conditions with widely recognized utility of CPU were identified as needing CPU only sometimes or rarely by many faculty. These results suggest that educational resources for CPU education may be lacking during the clinical medical school curriculum.

## A24 Clinical outcomes in the use of lung ultrasound for the diagnosis of pediatric pneumonias

### Angela Hua^1,2^, Sharon Kim^1^, James Tsung^1^

#### ^1^Department of Emergency Medicine, Icahn School of Medicine at Mount Sinai, New York City, USA; ^2^Department of Emergency Medicine, Northwell Health, New Hyde Park, USA

##### **Correspondence:** Angela Hua, e-mail: hua.angie@gmail.com

*Critical Ultrasound Journal* 2016, **8(Suppl 1)**:A24

**Purpose:** Previous data have demonstrated a significant reduction in CXR utilization (38.8 %) without missed pneumonia or other adverse outcomes when lung ultrasound was the initial imaging modality for evaluating suspected pediatric pneumonia. The purpose of this study was to assess the change in CXR utilization and impacts in clinical outcomes when using lung ultrasound as the initial imaging modality to evaluate suspected pediatric pneumonia.

**Methods:** We conducted a retrospective cohort study of pediatric emergency department (ED) patients aged 0–21 years presenting to an urban academic ED between March 2013 and January 2016, who received lung ultrasounds by experienced sonologists for the evaluation of suspected pneumonia. Primary outcome was the rate of CXR reduction. Secondary outcomes were rates of antibiotic use, admissions, and subsequent unscheduled healthcare visits (i.e., ED return visits).

**Results:** The charts of 341 subjects were reviewed. Of these, 116 (34 %) subjects were found to have normal ultrasounds, 115 (34 %) subjects had viral pneumonia, and 79 (23 %) subjects were diagnosed with bacterial pneumonia on ultrasound. A 77.7 % (265/341) reduction in CXR utilization was observed. Four out of 21 (19 %) chest X-rays were read as negative in patients who had lung ultrasounds demonstrating bacterial pneumonia. Twenty-six (26/79 = 33 %) of the ultrasound-diagnosed pneumonias were sub-centimeter in size.

**Conclusions:** The use of lung ultrasound first for the evaluation of suspected pediatric pneumonia showed a substantial reduction of CXR utilization by experienced sonologists. Additionally, this study showed that a substantial percentage of CXR were read as negative in patients who had lung ultrasounds demonstrating bacterial pneumonia. These findings suggest that lung ultrasound may be not only a safe but perhaps even better alternative than CXR for the diagnosis of pediatric pneumonias.

## A25 Effectiveness of ultrasound in hypotensive patients

### Isa Gunaydin^1^, Zeynep Kekec^1^, Mehmet Oguzhan Ay^2^

#### ^1^Department of Emergency Medicine, School of Medicine, Cukurova University, Adana, Turkey; ^2^Department of Emergency, Corum Education and Research Hospital, Hitit University, Corum, Turkey

##### **Correspondence:** Zeynep Kekec, e-mail: zkekec@cu.edu.tr

*Critical Ultrasound Journal* 2016, **8(Suppl 1)**:A25

**Purpose:** In this study, we aimed to investigate if early diagnosis and proper management can lessen morbidity and mortality in patients presenting with shock by utilizing bedside ultrasonographic scanning and RUSH (rapid ultrasound in shock) protocol.

**Methods:** This cross-sectional prospective study was conducted in the Department of Emergency Medicine of the Çukurova University Medical Faculty with the approval of the ethical committee, between August 1, 2014 and November 30, 2015. One hundred patients over the age of 18 and whose blood pressure was equal to or less than 90/60 mmHg were included in this study. Bedside ultrasonography and RUSH protocol were performed in each patient to assess the reasons of hypotension and shock in the emergency department (ED).

**Results:** Thirty-five female and 65 male patients were included in the study. The mean age of the patients was 57.12 ± 17.1. The complaints of the patients’ on admission were hematemesis or hematochesia (17 %), fatigue and decreased level of consciousness (13 %), dyspnea (13 %), decreased oral intake (12 %) and trauma (18 %). The most frequent findings on electrocardiography were tachycardia (57 %) and sinus tachycardia (48 %). The most common type of shock was hypovolemic shock (55 %) because 50 patients’ inferior vena cava indexes were under 1.5 cm. The most common reason of the shock was hemorrhage. Eighteen percent of the patients were found to have free fluid in the abdomen upon evaluation by FAST. Aortic aneurysm was seen in one patient and aortic dissection was seen in three patients. Cardiogenic shock was diagnosed in nine patients: four were due to arrhythmia and five were due to decompensated heart failure. Obstructive shock was diagnosed in nine patients (4 pericardial tamponade, 4 pulmonary thromboembolism and 1 pneumothorax). By the RUSH protocol, patients with hypovolemic shock (55 patients), distributional shock (19 patients), cardiogenic shock (8 patients), obstructive shock (6 patients) and complex shock (12 patients) were initially diagnosed. Poor clinical progression was seen in 60 % of our patients: 56 % of patients were transferred to intensive care unit and mortality occurred in 4 % of the patients in the ED. Good clinical progression was seen in the 40 % of our patients: 14 % of patients were discharged from the ED, while 26 % of them were hospitalized in different wards.

**Conclusions:** RUSH protocol is an early systematic approach and a practical and effective method to evaluate patients with hypotension and shock in the ED. It is useful in early diagnosis of hypotension and shock reasons by evaluating heart pump function, body volume condition, and vessel evaluation.

## A26 Moderate-to-severe left ventricular ejection fraction related to short-term mortality of patients with post-cardiac arrest syndrome after out-of-hospital cardiac arrest

### Jinjoo Kim, Jinhyun Kim, Gyoosung Choi, Dowon Shim

#### Gachon University Gil Medical Center, Incheon, Republic of Korea

##### **Correspondence:** Jinjoo Kim, e-mail: empearl@gilhospital.com

*Critical Ultrasound Journal* 2016, **8(Suppl 1)**:A26

**Purpose:** The aim of this study was to investigate the relationship between left ventricular ejection fraction (LVEF) and mortality and neurologic outcomes with post-cardiac arrest syndrome (PCAS) after out-of-hospital cardiac arrest (OHCA).

**Methods:** This is a retrospective cohort study. Patients with PCAS after OHCA admitted to intensive care unit between January 2014 and December 2015 were analyzed retrospectively.

**Results:** Totally 104 patients were enrolled in this study. The mean age of the patients was 54.4 ± 15.3 years and the number of male patients was 75 (72.1 %). Arrest of cardiac origin was reported in 55 (52.9 %) patients. Bedside echocardiography LVEF <45, 45–55, >55 % were 39 (37.5) %, 18 (17.3) %, 47 (45.2) %, respectively. In multivariate analysis, severe LV dysfunctions (LVEF <45 %) were statistically significantly related to 7-day mortality (odds ratio 3.02, 95 % confidence interval 1.01–9.0, *p* 0.047).

**Conclusions:** In this study, moderate-to-severe LVEF within 48 h after return of spontaneous circulation was significantly related to 7-day short-term mortality of patients with PCAS after OHCA. Clinicians would treat myocardial dysfunction actively, and further studies are needed.

## A27 Usefulness of abdominal ultrasound for acute pyelonephritis diagnosis after kidney transplantation

### Ji-Han Lee

#### Chungbuk National University Hospital, Cheong-Ju, South Korea

##### **Correspondence:** Ji-Han Lee, e-mail: capcloud80@hanmail.net

*Critical Ultrasound Journal* 2016, **8(Suppl 1)**:A27

**Purpose:** If kidney transplant patients have localization of pain to the right lower quadrant abdomen, primary physician requires differential diagnosis with appendicitis and transplanted kidney infection. But the use of contrast APCT can be harmful due to kidney toxicity. Fortunately, transplanted kidney is easy to access because they are exposed to the abdominal cavity than normal retroperitoneal kidneys. So one can check sono-guided tenderness and make the diagnosis easy using ultrasound.

**Method:** Position the ultrasound probe at the position of pain and scan internal organs. Make sure that pain or discomfort is caused by applying pressure over ultrasound probe. This maneuver is probably more accurate than palpation with the hand because the ultrasound transducer can confirm that the transplanted kidney is being pushed when the patient experiences the pain.

**Result:** Kidney transplant patients with right lower quadrant abdominal pain requires differential diagnosis with appendicitis and transplanted kidney infection. Using ultrasound in renal transplant patients is useful and reduces the use of diagnostic contrast CT.

**Conclusion:** In renal transplant patients with UTI, diagnosis using ultrasound is easy and harmless.

## A28 Lung ultrasound for assessing fluid tolerance in severe preeclampsia

### Jana Ambrozic^1^, Katja Prokselj^1^, Miha Lucovnik^2^

#### ^1^Department of Cardiology, University Medical Center Ljubljana, Ljubljana, Slovenia; ^2^Division of Obstetrics and Gynecology, Department of Perinatology, University Medical Center Ljubljana, Ljubljana, Slovenia

##### **Correspondence:** Miha Lucovnik, e-mail: miha.lucovnik@kclj.si

*Critical Ultrasound Journal* 2016, **8(Suppl 1)**:A28

**Purpose:** Assessment of extravascular lung water (EVLW) is fundamental in the management of patients with severe preeclampsia. Due to increased capillary permeability associated with this disorder, even small fluid excesses can lead to pulmonary edema. Poor fluid management of preeclamptic patients has been shown to increase the risk of severe maternal morbidity and even mortality. The purpose of this study was to evaluate EVLW lung ultrasound measurements in patients with severe preeclampsia before delivery as well as in the first days postpartum.

**Methods:** The study population consisted of patients consecutively admitted at a single level III referral institution with the diagnosis of preeclampsia with severe features between April 2015 and February 2016. Severe features of preeclampsia were defined using the American College of Obstetricians and Gynecologist Task Force on Hypertension in Pregnancy recommendations. Lung ultrasound was performed according to a systematic protocol in supine patients. The echo comet score (ECS) was obtained by the 28-rib interspaces technique dividing the chest wall in 12 areas on the left (from the second to the fourth intercostal space) and 16 areas on the right (from the second to the fifth intercostal space) anterior and lateral hemithorax. Statistical comparison between ECS before delivery vs. within 24 h post-delivery vs. 4 days post-delivery was performed using repeated measures ANOVA. A *p* value of <0.05 was considered significant.

**Results:** Seventeen women with preeclampsia with severe features were included. Echo comet score measured before delivery was significantly higher (mean 28, range 12–70) vs. 24 h post-delivery (mean 23, range 3–51), vs. 4 days post-delivery (mean 23, range 0–39) (*p* = 0.03).

**Conclusions:** EVLW is significantly increased in severe preeclampsia and it decreases rapidly following delivery. Lung ultrasound allows noninvasive assessment of EVLW in preeclamptic patients and can be used to guide fluid as well as diuretic therapy in the peripartum period.

## A29 Optic nerve sheath ultrasound in severe preeclampsia

### Gabrijela Brzan Simenc^1^, Jana Ambrozic^2^, Miha Lucovnik^1^

#### ^1^Division of Obstetrics and Gynecology, Department of Perinatology, University Medical Center Ljubljana, Ljubljana, Slovenia; ^2^Department of Cardiology, University Medical Center Ljubljana, Ljubljana, Slovenia

##### **Correspondence:** Miha Lucovnik, e-mail: miha.lucovnik@kclj.si

*Critical Ultrasound Journal* 2016, **8(Suppl 1)**:A29

**Purpose:** Neurological complications of preeclampsia are due to failure of cerebrovascular autoregulatory mechanisms in the setting of increased blood pressure and endothelial dysfunction. Signs compatible with increased intracranial pressure (ICP) on brain imaging have been reported in cases of severe preeclampsia. Ultrasound measurements of optic nerve sheath (ONS) have been shown outside pregnancy to correlate with raised ICP. There are limited data on the usefulness of this noninvasive technique in severe preeclamptic patients. The purpose of this study was to compare ONS diameter measurements in patients with severe preeclampsia before delivery to those performed in the first days postpartum.

**Methods:** The study population consisted of patients consecutively admitted at a single level III referral institution with the diagnosis of preeclampsia with severe features between April 2015 and February 2016. Severe features of preeclampsia were defined using the American College of Obstetricians and Gynecologist Task Force on Hypertension in Pregnancy recommendations. Transverse plane ONS diameter measurements were performed using a 7.5 MHz linear probe. Three measurements were made for each ONS and mean values analyzed. Statistical comparison between ONS diameter before delivery vs. within 24 h post-delivery vs. 4 days post-delivery was performed using repeated measures ANOVA. A *p* value of <0.05 was considered significant.

**Results:** Seventeen women with preeclampsia with severe features were included. ONS diameters were significantly larger before delivery (mean 6.1 mm, range 5.5–7.5 mm) vs. 24 h post-delivery (mean 6.0 mm, range 5.3–7.0 mm), vs. 4 days post-delivery (mean 5.5 mm, range 4.4–6.5 mm) (*p* = 0.01). Proportions of patients with ONS diameter above 5.8 mm (associated with increased ICP) were 65 % before delivery, 59 % within 24 h post-delivery, and 24 % 4 days post-delivery.

**Conclusions:** ONS diameters are compatible with increased ICP in up to 65 % of severe preeclamptic patients before delivery and decrease rapidly in the first days postpartum. Ultrasound ONS measurements may detect patients with neurological complications of preeclampsia, which can help identify optimal time for delivery by balancing fetal/neonatal risks and risks of adverse maternal neurological outcome.

## A30 Focused echocardiography monitoring in the postoperative period for non-cardiac patients

### Asta Mačiulienė^1^, Almantas Maleckas^2^, Algimantas Kriščiukaitis^3^, Vytautas Mačiulis^1^, Andrius Macas^1^

#### ^1^Department of Anaesthesiology, Lithuanian University of Health Sciences Kaunas Clinics, Kaunas, Lithuania; ^2^Department of General Surgery, Lithuanian University of Health Sciences Kaunas Clinics, Kaunas, Lithuania; ^3^Department of Physics, Mathematics and Biophysics, Lithuanian University of Health Sciences, Kaunas, Lithuania

##### **Correspondence:** Andrius Macas, e-mail: Andrius.Macas@kaunoklinikos.lt

*Critical Ultrasound Journal* 2016, **8(Suppl 1)**:A30

**Background and aim**: Various methods were investigated for the evaluation of intravascular fluid status. Inferior vena cava (IVC) index is a reliable measure to evaluate fluid responsiveness in patients under mechanical ventilation. However, IVC index for spontaneously breathing patients is debatable as different authors show controversial results. The aims of the study were to evaluate the feasibility and reliability of focused assessed transthoracic echocardiography in postoperative noncardiac patients and to compare different methods of focused echocardiographic data for defining hypovolemia.

**Methods:** A prospective pilot study was carried out in the Department of Anesthesiology, the Hospital of Lithuanian University of Health Sciences Kauno Klinikos. Hypovolemia was evaluated by the list of clinical signs: thirst, dry mucous membranes, such as the mouth, loss of skin elasticity, tachypnea, capillary refill time, urinary output, heart rate, mean arterial pressure, and CVP (mmHg) when available. Patients were classified as hypovolemic and nonhypovolemic. Another independent investigator performed echocardiographic evaluation using FATE protocol. All focused echocardiographic measurements were performed by the same trained anesthesiologist. The IVC index and mitral E and A waves were measured, and the global echocardiographic view was evaluated.

**Results:** Fifty-six patients, including 23 (42.2 %) men and 33 (58 %) women, who underwent abdominal surgery were included in this study. The mean age was 61.68 (58.02–65.33) years. ASA physical status grade II was found in 31 (55.4 %) and grade II in 21 (44.6 %), respectively. The proportion of adequate views was significantly lower for the subcostal view and IVC view while trying to get the image from subcostal long-axis view (*p* < 0.001). An adequate subcostal four-chamber view was obtained in 40 (71.4 %) patients, adequate IVC images from subcostal long-axis view were obtained in 39 (69.6 %) patients. The lower success rate of obtaining an adequate subcostal view was associated with experienced moderate postoperative pain (*p* = 0.004). The problems related with operation type which had an influence on image quality were postoperative wounds (bandages after laparotomy) and intra-abdominal gases after laparoscopy (*p* < 0.001). Hypovolemia by clinical signs was identified in 8 (14.3 %) patients compared to 16 (28.6 %) patients classified as hypovolemic by basic echocardiographic view, 14 (25 %) by IVC variability index, and 20 (35.7 %) by E/A ratio. The incidence of hypovolemia by clinical signs was significantly lower compared with the identification of hypovolemia by echocardiographic data (*p* = 0.009).

**Conclusions**: In most cases, it is possible to obtain good-quality echocardiographic images that are suitable for interpretation and decision making in patients after abdominal surgery. Identification of hypovolemia is significantly higher by echocardiographic data (IVC index, global echo view, mitral E/A ratio) compared with clinical signs. However, there are different limitations for these parameters. We suggest not to base decision making on one of them. While these parameters are quite easily measured by trained doctor, the whole complex should be evaluated.

## A31 POCUS-guided paediatric upper limb fracture reduction: algorithm, tricks, and tips

### Sharad Mohite

#### Department of Paediatrics, Children’s Emergency, National University Hospital, Singapore, Singapore

##### **Correspondence:** Sharad Mohite, e-mail: mohite_sharad_arvindrao@nuhs.edu.sg

*Critical Ultrasound Journal* 2016, **8(Suppl 1)**:A31

Ultrasound-guided closed fracture reduction is not a new concept. However, with recent thrust on training and use of point-of-care ultrasound (POCUS) by Paediatric Emergency physicians, it has become imperative to highlight this relatively simple application of POCUS to the emergency physicians. In children, fractures of the forearm are most common type of fractures. This is followed by fractures of the humerus and phalanges. POCUS-guided closed manipulation and reduction (M&R) of these fractures is recommended as it would reduce the need for repeated radiographs, sedation, and admissions due to unsatisfactory reduction or alignment. However, it is important to understand certain basic differences between conventional M&R by digital palpation and POCUS-guided M&R. In this respect, I would like to suggest some techniques to minimize failures and improve the successful outcomes. Each fracture has different anatomy when seen on X-rays from that seen on ultrasonography, hence while reducing fractures this needs to be taken into consideration. With three examples of upper limb fractures in children (distal radius, Salter–Harris Type II, displaced supracondylar fracture, and displaced fracture phalanx), I would like to explain how to reduce each of them. I would also like to propose an algorithm for POCUS-guided fracture reduction. Lastly, I would like to share some practical tricks and trips while performing fracture reductions. This abstract needs to be considered for oral presentation only.

## A32 Point-of-care lung ultrasound: a good diagnostic tool for pneumonia in a septic patient

### Zoltan Narancsik, Hugon Možina

#### ^1^Emergency Medical Unit, Faculty of Medicine Ljubljana, University Medical Centre Ljubljana, Ljubljana, Slovenia

##### **Correspondence:** Zoltan Narancsik, e-mail: zoltan.narancsik@gmail.com

*Critical Ultrasound Journal* 2016, **8(Suppl 1)**:A32

**Purpose:** Patients with severe sepsis have high mortality rates. Early diagnosis and treatment provide better outcomes. Diagnosis of the infectious focus within the first hour is important in the selection of effective intravenous antimicrobials. The most common origin of infections that develop into sepsis is pneumonia. In addition to suggestive clinical and laboratory features, a demonstrable infiltrate by chest radiograph (CXR) is commonly required for the diagnosis of pneumonia. Recent studies show that lung ultrasound (LUS) is highly effective, perhaps even better than CXR in evaluating and differentiating pulmonary conditions such as pneumonia and cardiogenic pulmonary edema. We present a case of a patient with severe sepsis in whom the usual diagnostic modalities failed to discover the infectious focus; however, using LUS we discovered that the patient had pneumonia.

**Methods:** A 67-year-old patient with COPD and an artificial aortic valve was admitted to our emergency department (ED) complaining of weakness, fever, chills, and dyspnea, lasting for 3 days, with gradual worsening. On examination, she was ill-looking, somnolent, and had difficulty talking. She was febrile, normotensive, with tachycardia and tachypnoea, and had oxygen saturation of 88 on 100 % inhaled oxygen via non-rebreathing mask. Chest examination revealed inspiratory crackles in both basal lung fields with prolonged expiratory sounds. Laboratory tests suggested systemic inflammation with decreased kidney function. Arterial blood gas analysis revealed acute respiratory failure and acidosis. The CXR was unremarkable; however, bedside LUS revealed focal B lines and the presence of subpleural lung consolidation in the right anterior lung area, confirming pneumonia, excluding pulmonary edema, and allowing for intensive fluid resuscitation and rational antibiotic selection.

**Conclusions:** Our case presentation illustrates the usefulness of bedside LUS in fast and accurate diagnosis of pneumonia in a septic patient where CXR was inconclusive. This bedside diagnostic tool is especially useful in the ED.

**Informed consent** This study was conducted in accordance with the ethical standards dictated by applicable law. Informed consent was obtained from each owner for enrolment in the study and the inclusion in this article of information that could potentially lead to their identification.

## A33 A case of undergraduate POCUS (r)evolution

### Sara Nikolić^1^, Jan Hansel^2^, Rok Petrovčič^1^, Una Mršić^1^, Gregor Prosen^3^

#### ^1^Faculty of Medicine, University of Maribor, Maribor, Slovenia; ^2^Landspítali University Hospital, Reykjavík, Iceland; ^3^Department of Emergency Medicine, University Clinical Center Maribor, Maribor, Slovenia

##### **Correspondence:** Gregor Prosen, e-mail: gregorprosen@gmail.com

*Critical Ultrasound Journal* 2016, **8(Suppl 1)**:A33

**Purpose:** Ultrafest is a single-day intensive course in point-of-care ultrasound (POCUS) for undergraduate medical students. Our team adapted the concept from University of California Irvine, organizing the first European iteration in January 2015, followed by courses in May and November. The objectives were to introduce undergraduate medical students to POCUS, to investigate the viability of a flipped classroom model for teaching theoretical POCUS concepts, and to motivate students for further postgraduate POCUS training.

**Methods:** Students attending Ultrafest (*n* = 134) were provided video lectures a month in advance to study basic POCUS concepts. The seven selected videos were recorded by UCI and lasted 4 h altogether. Theoretical knowledge was tested on the day of the event with a pretest consisting of 15 single best answer multiple-choice questions of mixed format (recall questions, clinical vignettes with pictures). The event consisted of 2 h of didactics and 6 h of hands-on training, conducted by certified clinicians. Ultrafest was concluded by students completing a 5-point Likert scale questionnaire and a posttest.

**Results:** Students were successful at pretest with the mean result of 13.70 out of 15 points. There was a statistically significant difference between the results of students who reviewed 0–4 videos (mean score 11.86, *n* = 21) and students who reviewed 5–7 videos (mean score 14.05, *n* = 113) (*p* < 0.0001). On the post-event survey, students agreed that video lectures were appropriate for achieving learning outcomes (4.81), considered them useful for further studies and clinical practice (4.82), and mostly preferred video to classic lectures (4.16). They were highly motivated to further improve their POCUS skills (4.93), feel confident to perform a bedside exam (4.24), find POCUS highly relevant for their further studies (4.90), and feel that more time should be allotted to teaching US skills (3.72).

**Conclusions:** The flipped classroom approach utilizing video lectures is a valid modality for teaching theoretical POCUS concepts to undergraduate medical students. Students see it as a welcome augmentation to classic lectures. We find that a single-day intensive POCUS course is an attractive, motivating, and effective first step in introducing POCUS basics at the undergraduate level. Our study was limited by uneven research groups due to opportunity sampling.

## A34 The Graz Summer School for ultrasound: from first contact to bedside application: three-and-a-half-day undergraduate ultrasound training: résumé after two years of continuous development

### Simon Orlob^1,2^, Markus Lerchbaumer^1,2^, Niklas Schönegger^1,2^, Reinhard Kaufmann^1,2^

#### ^1^Medical University Graz, Graz, Austria; ^2^Sono4You Graz, Graz, Austria

##### **Correspondence:** Simon Orlob, e-mail: simon.orlob@medunigraz.at

*Critical Ultrasound Journal* 2016, **8(Suppl 1)**:A34

**Purpose:** Ultrasound, and especially the focused, bedside application, becomes a common tool in good daily practice. However, most medical schools do not represent this development in their undergraduate trainings yet. Our group designed a three-and-a-half-day multicenter undergraduate course to offer an introduction into the examination modality of point-of-care ultrasound.

**Methods:** In this emergency ultrasound course, three focused ultrasound protocols (FAST, RUSH, and Lung sonography + DVT) are taught in their clinical context. Each protocol is introduced after having discussed the differential diagnosis and the clinical pathways of a traumatized, hypotensive, and dyspneic patient, respectively. While the lectures are given by experienced physicians, the hands-on workshops are held by student peer teachers in small groups (5 participants: 2 peer teachers). All lectures are made open accessible via YouTube-Livestream. To facilitate viewers’ interaction, a parallel video-conference is held. The last day is concluded by a ‘sonography ward round’ giving every student the opportunity to gain first experiences in bedside application in ICU and normal ward patients.

**Results:** The program covers 8.25 h of lectures and 10.25 h of hands-on training, resulting in 125 min of active scanning per participant. Asked for their curricular training, just 10 % of the participants replied that they are trained very good in sonography, and 40.8 and 44.9 % stated to be trained poor or even very poor, respectively. Regarding the didactical approach, 93.9 % of the students stated that the didactical concept is very good. 60 students had a spot in Graz. Students at the universities of Berlin and Heidelberg received the lectures via web-conference, providing hands-on sessions locally.

**Conclusions:** The Summer School not just teaches image acquisition and interpretation, but rather puts the ultrasound examination in a clinical context and finalises with bedside application of the course content. With 60 participants and provision of 15 ultrasound machines, the local maximum capacity is almost reached. The conjunction of lectures via Livestream and local hands-on sessions generates more spots in total with minimized efforts organizing the course at a remote location. Incorporating new broadcasting techniques increased the outreach of the course by manyfold, gaining awareness for the importance of bedside ultrasound, until now just poorly represented in undergraduate training.

## A35 Usefulness of point-of-care ultrasound in the emergency room in a patient with acute abdominal pain

### Alberto Oviedo-García, Margarita Algaba-Montes, Mayra Patricio-Bordomás

#### ^1^Emergency Department, Hospital de Valme, Universidad de Sevilla, Seville, Spain

##### **Correspondence:** Alberto Oviedo-García, e-mail: albertoaog1972@hotmail.com

*Critical Ultrasound Journal* 2016, **8(Suppl 1)**:A35

**Purpose:** To demonstrate the utility of emergency ultrasound in a patient with acute abdominal pain. Bedside ultrasound is being used with increasing frequency by emergency physicians as goal-directed examinations meant to answer specific questions. In patients with abdominal pain, ultrasound can be used to rapidly determine the presence or absence of an abdominal aortic aneurysm, gallstones, hydronephrosis, intra-abdominal hemorrhage, etc. The use of ultrasound by emergency physicians in Spain is progressively rising, more and more emergency departments have ultrasound machines, and more and more doctors are trained in its use in emergencies settings.

**Methods:** This is a case study of the diagnosis of a pneumoperitoneum using ultrasound at the bedside of the patient in the emergency room. We used a Sonosite M-Turbo, with convex probe C60e/5-2 MHz.

**Results:** An 81-year-old male, with good quality of life, attended the emergency room with abdominal pain, 2-h evolution, feeling very unwell, very restless, sweaty, hypotensive, tachycardic, tachypneic, and desaturating, with overall poor perfusion peripheral and peritonism data on abdominal palpation. The emergency physician performed an ultrasound at the bedside identifying free peritoneal fluid with echoes inside (air bubbles), along with a peritoneal hyperechoic line that caused reverberation artifacts (similar to lines B of lung ultrasound) and location changed by modifying the position of the patient, data described in the literature with pneumoperitoneum by viscera perforation. Given the poor clinical condition, after intensive hemodynamic treatment, the patient was taken to the operating room where a perforated duodenal ulcer was observed.

**Conclusions:** Ultrasound is not sufficiently used by emergency physicians in the detection of pneumoperitoneum and its associated signs, but it is imperative that emergency physicians be familiar with ultrasound findings associated with an acute abdomen, such as the detection of an abdominal aortic aneurysm, intra-abdominal hemorrhage, or perforation of a hollow organ, among others. The authors suggest that ultrasound at the bedside of critically ill patients with acute abdomen and suspected pneumoperitoneum can be a very useful tool when poor hemodynamic situation makes the transfer of the patient to the radiology department difficult, as in the previous case presented.

**Informed consent** This study was conducted in accordance with the ethical standards dictated by applicable law. Informed consent was obtained from each owner for enrolment in the study and the inclusion in this article of information that could potentially lead to their identification.

## A36 Use of bedside ultrasound in a critically ill patient. A case report

### Alberto Oviedo-García, Margarita Algaba-Montes, Mayra Patricio-Bordomás

#### Emergency Department, Hospital de Valme, Universidad de Sevilla, Seville, Spain

##### **Correspondence:** Alberto Oviedo-García, e-mail: albertoaog1972@hotmail.com

*Critical Ultrasound Journal* 2016, **8(Suppl 1)**:A36

**Purpose:** To demonstrate the usefulness of clinical ultrasound in the hands of emergency physicians with critically ill septic patients. Emphysematous cholecystitis is a rare entity that represents 1 % of all cholecystitis, with indistinguishable symptoms, but with a negative prognosis (25 % mortality) and a greater number of complications; it, therefore, requires early diagnosis, which allows adequate management as soon as possible, in order to prevent a fatal outcome.

**Methods:** This is a case study of the diagnosis of an emphysematous cholecystitis using point-of-care ultrasound performed by emergency physician. We have a Sonosite M-Turbo, with convex probe C60e/5-2 MHz.

**Results:** A 71-year-old male was admitted to the emergency room with the right upper quadrant pain which started several days before, accompanied with asthenia, vomiting, and fever. On examination, he was affected, hypotensive, tachycardic, sweating, and febrile. The clinical situation of this patient with sepsis revealed tenderness in the right upper quadrant. Given the serious clinical condition, the emergency physician made a point-of-care ultrasound scan that showed a distended gallbladder, with thickened walls and hyperechoic areas on the anterior wall, which caused posterior acoustic shadowing, as well as biliary sludge inside; all of this was compatible with emphysematous cholecystitis. Intensive hemodynamic support measures and early empirical antibiotic therapy were initiated, and urgent surgery was recommended. The evolution was favorable without further complications and he was discharged from the hospital 8 days after admission.

**Conclusions:** Ultrasound is the diagnostic technique of choice for the diagnosis of acute cholecystitis. It is safe, fast, and accurate, with a sensitivity of 90–95 and a specificity of 70–90 %. The authors believe that the use of abdominal ultrasound in the emergency room should be extended to all emergency physicians, because it allows a quick and versatile diagnosis, as well as a suitable treatment for early-onset to severe patients, as in the case presented. This is vital for a better prognosis and good evaluation of our patients, avoiding serious complications and providing greater clinical patient safety.

**Informed consent** This study was conducted in accordance with the ethical standards dictated by applicable law. Informed consent was obtained from each owner for enrolment in the study and the inclusion in this article of information that could potentially lead to their identification.

## A37 Diagnostic yield of clinical echocardiography for the emergency physician

### Alberto Oviedo-García, Margarita Algaba-Montes, Mayra Patricio-Bordomás

#### ^1^Emergency Department, Hospital de Valme, Universidad de Sevilla, Seville, Spain

##### **Correspondence:** Alberto Oviedo-García, e-mail: albertoaog1972@hotmail.com

*Critical Ultrasound Journal* 2016, **8(Suppl 1)**:A37

**Purpose:** To demonstrate the utility of clinical echocardiography in a febrile patient. Bedside ultrasound is being used with increasing frequency by emergency physicians as goal-directed examinations meant to answer specific questions. The use of ultrasound by emergency physicians in Spain is progressively rising, more and more emergency departments have ultrasound machines, and more and more doctors are trained in its use in emergencies settings.

**Methods:** This is a case study of the diagnosis of an infective endocarditis using ultrasound at the bedside of the patient in the emergency room. We used a Sonosite M-Turbo, with P21 probe 1–5 MHz.

**Results:** A 58-year-old female attended the emergency room with fever of several weeks of evolution; on arrival, she complained of difficulty in breathing, looked unwell, and was febrile, tachypneic, and tachycardic. Her vital signs were as follows: temperature, 38,4 °C; blood pressure, 110/58 mmHg; respiratory rate, 30 breaths per minute; room air oxygen saturation, 88 %; and heart rate, 140 beats per minute. The emergency physician performed bedside echocardiography that showed a large mass in the anterior mitral valve, with pendulum movement causing moderate mitral regurgitation. Empirical antibiotic therapy started early, later confirmed the findings by a formal transesophageal echocardiography, initially ruled out urgent repair surgery, and being admitted to the Infectious Diseases Department.

**Conclusions:** According to current guidelines, echocardiography should be performed in all patients with moderate-to-high suspicion of infective endocarditis, allowing the detection of valvular vegetations, regurgitations, assess the hemodynamic status of the patient, etc. being transthoracic the first to perform in patients with suspected infective endocarditis, resulting positive study if it detects vegetation, but their absence does not exclude the diagnosis. The case shows benefits that echocardiography may have in emergency doctor hands that combine clinical and ultrasonographic data, reaches an accurate diagnosis and appropriate antibiotic treatment begins immediately.

**Informed consent** This study was conducted in accordance with the ethical standards dictated by applicable law. Informed consent was obtained from each owner for enrolment in the study and the inclusion in this article of information that could potentially lead to their identification.

## A38 Focused cardiac ultrasound in early diagnosis of type A aortic dissection with atypical presentation

### Chun-I Pan, Hsiu-Yung Pan, Chien-Hung Wu

#### ^1^Emergent Department, Kaohsiung Chang Gung Memorial Hospital, Kaohsiung City, Taiwan

##### **Correspondence:** Chun-I Pan, e-mail: vitrobone@gmail.com

*Critical Ultrasound Journal* 2016, **8(Suppl 1)**:A38

**Purpose:** To diagnose the chest pain induced by type A aortic dissection in the elderly, early by focused cardiac ultrasound.

**Methods:** An elder man presented with chest pain, suspecting of acute myocardial infarction. Focused cardiac ultrasound was performed by emergency physician for evaluation of regional wall motion abnormalities moving intimal flap within dilated aortic root. Then a cardiovascular surgeon was consulted, and the patient received emergent surgical repair and was discharged from ward 2 weeks later with no adverse outcomes reported.

**Results:** Acute dissection of the thoracic aorta is challenging to diagnosis and imaging studies as magnetic resonance angiography, computed tomographic angiography, and multiplane transesophageal echocardiography should be performed for definite diagnosis. However, these imaging tools are not always available in local medical department. And our case revealed that focused transthoracic echocardiography offers dynamic evaluation, which changes the therapy timely and safely.

**Conclusions:** Emergency physicians could detect significant findings that are not noticed on clinical evaluation by performing focused cardiac ultrasound and offer the most appropriate treatment for the patients as in our patient.

## A39 Detection of imperforated hymen by point-of-care ultrasound

### Hsiu-yung Pan, Chia-Te Kung

#### Emergency Department, Kaohsiung Chang Gung Memorial Hospital, Kaohsiung City, Taiwan

##### **Correspondence:** Hsiu-yung Pan, e-mail: gettingfat720@gmail.com

*Critical Ultrasound Journal* 2016, **8(Suppl 1)**:A39

**Purpose:** To use the point-of-care ultrasound (POC-US) for abdominal pain etiology survey at ED.

**Methods:** POC-US was employed to study the cause of abdominal pain in a premenarche girl.

**Results:** Heteroechoic mass over pelvis was revealed. Imperforated hymen was suspected clinically. Abdominal CT was performed under gynecologist suggestion with hematometra and hematocolpos shown.

**Conclusion:** Imperforated hymen is an obstructive lesion of the female genital tract and its incidence approximates 1 in 1000–2000 females. At birth, the newborn may have a bulging translucent yellow-gray mass at the vaginal introitus due to mucocolpos from vaginal secretions stimulated by estradiol. The mucus will be reabsorbed and most cases are asymptomatic until menarche. After menarche, the adolescent girls with imperforated hymen may present with cyclic abdominal or pelvic pain, amenorrhea, and difficulty with urination or defecation. With cyclic menstruation, the vaginal canal distended greatly and the cervix may dilate, which leads to the formation of hematometra and hematocolpos. Moreover, retrograde menstruation may lead to the development of endometriosis. Inspection of the external genital organs is indicated but is often deferred by the parents and the patient. We approached her with POC-US and detected hypoechoic masses in the pelvis which were compatible with hematometra and hematocolpos. For adolescent girl without sexual experience, inspection of the genital tract is embarrassing especially if the examination is performed by male physicians and doctors other than gynecologist. POC-US provides noninvasive and radiation-free means for diagnosis and helps in making treatment plan in this patient. Abdominal pain is a common emergency department (ED) complaint but is often diagnostically challenging. Uncertainty and diagnostic possibilities can be even greater in females, besides the fact that the extent of differential diagnosis varies between different age groups. Here, we describe an adolescent female with abdominal pain owing to imperforated hymen, which was disclosed by POC-US.

## A40 Developing a point-of-care ultrasound curriculum for pediatric nurse practitioners practicing in the pediatric emergency department

### Sarah Pasquale, Stephanie J. Doniger, Sharon Yellin, Gerardo Chiricolo

#### ^1^New York Methodist Hospital; Brooklyn, New York, USA

##### **Correspondence:** Sarah Pasquale, e-mail: sarahpasquale@gmail.com

*Critical Ultrasound Journal* 2016, **8(Suppl 1)**:A40

**Study objectives:** Pediatric point-of-care ultrasound (POCUS) is becoming an important diagnostic aid and procedural adjunct. While POCUS has had a long history being successfully taught to physicians and even medical students, few studies have involved the specific training of Nurse Practitioners. We aimed to develop and implement a novel, tailored POCUS curriculum based on the specific needs of pediatric nurse practitioners (PNPs) practicing in the pediatric emergency department (PED). We also sought to assess the PNPs’ knowledge retention and proficiency of POCUS over time.

**Methods:** This is a prospective pilot study conducted at a dedicated PED with 27,000 pediatric visits per year.

This study consisted of five phases:

1. The recruitment of three PNPs and the completion of a pre-course assessment which included the applications they felt would impact their practice most. We reviewed the radiology-performed ultrasounds they ordered over the preceding 6 months. Both helped determine the curriculum content;

2. The administration of an 8-hour course, which involved didactic lectures followed by a hands-on session. Identical pre- and post-course written examinations were administered;

3. Dedicated one-on-one scanning sessions with one of the Pediatric Emergency Ultrasound faculty;

4. Six-month period, during which the NPs performed POCUS examinations. These were all recorded and reviewed for Quality Assurance;

5. A 6-month follow-up examination and survey.

**Results:** The 3 NPs had differing levels of experience in the PED (6 months to 4 years). None had completed any prior POCUS training and rated their overall comfort level as 1–2 on a Likert scale. All of the PEM NPs rated their specific interest in learning POCUS and its potential positive impact on their clinical practice between 3 and 4 on a Likert scale. They ranked Ob/Gyn and appendicitis as the most important and focused cardiac as the least important to learn.

The performance on the pre-, post-, and 6-month follow-up examinations varied. For the pre-course test, scores were (PNP1, PNP2, PNP3) 73, 73, and 55 %. For the posttest, the scores were 73, 77, and 64 %. For the 6-month follow-up test, the scores were 7, 86, and 73 %. During the 6-month period, a total of 46 ultrasound examinations were performed (PNP1–21, PNP2–4, PNP3–21). Collectively, the PNPs performed POCUS examinations with an accuracy of 65 % (30/46). Only those scans that are considered to be technically accurate were assessed, which resulted in a 100 % sensitivity and specificity.

**Conclusions:** Pediatric NPs can be effectively taught specific applications of POCUS pertinent to their clinical practice.

## A41 Use of transthoracic echocardiography in emergency setting: patient with mitral valve abscess

### Maja Potisek, Borut Drnovšek, Boštjan Leskovar

#### Internal Medicine Department, General Hospital Trbovlje, Trbovlje, Slovenia

##### **Correspondence:** Maja Potisek, e-mail: maja.potisek@gmail.com

*Critical Ultrasound Journal* 2016, **8(Suppl 1)**:A41

**Purpose:** Through case report, we want to present that echocardiography is the most useful test in diagnosing heart failure and its etiology in acute settings.

**Results:** A 57-year-old female with no previously known heart disorder was admitted to our ward because of acute enterocolitis. Infectious parameters became elevated, empiric therapy with amoxicillin was started, but no infectious agent was isolated. At first, she responded well, later she became disorientated, taciturn, but remained afebrile, with low infectious parameters. Head CT scan showed no pathology. Due to suspected delirium or depressive disorder, consultant psychiatrist advised transfer to psychiatric clinic, where she became febrile, with high infectious parameters. Purulent meningitis was diagnosed after temporary admission to Infectious Diseases Clinic, and causative microorganism was not isolated. After few days of antibiotic therapy (ampicillin, cefotaxime), she became lucid but soon febrile again, with symptoms and signs of heart failure. Her condition deteriorated rapidly, and pulmonary edema persisted despite diuretics. We performed urgent bedside transthoracic echocardiography to define the cause:

• ventricles: normal size and systolic function, no segmental contractility disorders, LVEF 84;

• aortic, tricuspid, pulmonary valve: morphologically and functionally normal;

• mitral valve: posterior leaflet hyperechogenic, deformed on level of P3 and partially P2; suspected vegetation on posterior leaflet with prolapse and minor flail on P3 level; anechogenic area (2.5 × 2.2 cm) at posteromedial part of mitral annular abscess; severe mitral regurgitation, eccentric jet towards the anterolateral wall of left atrium. She was urgently transferred to cardiovascular surgery unit, where infective endocarditis (IE) was confirmed with transesophageal echocardiography. Mitral valve replacement was performed, and abscess cavity was cleaned.

**Conclusions:** The diagnosis of IE is based on modified Duke criteria, which in our case were not fulfilled. Transesophageal echocardiography is the technique of choice for diagnosis of IE, but in many cases transthoracic echocardiography is sufficient. Major diagnostic criteria are vegetation, abscess/pseudoaneurysm, and new dehiscence of prosthetic valve. Sensitivity of transthoracic echocardiography for diagnosis of vegetations and abscesses is 70 and 50 %, and that of transesophageal echocardiography is 96 % and 90 %, respectively.

**Informed consent** This study was conducted in accordance with the ethical standards dictated by applicable law. Informed consent was obtained from each owner for enrolment in the study and the inclusion in this article of information that could potentially lead to their identification.

## A42 A young man with syncope

### Fatemeh Rasooli, Maryam Bahreini

#### ^1^Emergency Medicine Department, Tehran University of Medical Sciences, Tehran, Iran

##### **Correspondence:** Fatemeh Rasooli, e-mail: fa.rasooli2@gmail.com

*Critical Ultrasound Journal* 2016, **8(Suppl 1)**:A42

**Purpose:** Syncope is a transient loss of consciousness that occurs when the blood pressure is low and the brain oxygen is insufficient. The most important causes of syncope include various cardiac, neurologic, and metabolic disorders or drug side effects. Obstructive, ischemic, or conductive heart diseases may cause syncope. In this case, syncope occurred due to atrial myxoma. Bedside ultrasonography was very helpful to achieve diagnosis and fast decision making in this patient.

**Methods:** A 27-year-old man presented to the emergency department with history of syncope, weakness, and severe fatigue. He had no complaint of chest pain or dyspnea. Nausea and non-bloody vomiting occurred once. Past medical and habitual history were negative. The patient mentioned two previous episodes of syncope. Vital signs were a blood pressure of 129/75 mmHg, a heart rate of 75 beats per minute, a respiratory rate of 18 breath per minute, and the oxygen saturation in room air of 99 %. He was awake but confused. Jugular vein was normal. A loud S1 was detected in heart auscultation. Respiratory and gastrointestinal examinations were normal. The patient complained of generalized symmetrical weakness although other neurological findings were normal.

**Results:** Laboratory findings were normal except for mild leukocytosis. Electrocardiography showed normal sinus rhythm and axis. All segments and intervals were in normal range. Interestingly, an obvious variability in heart rate was noticed on cardiac monitor, which was changing by the patient position that was a guide to the use of bedside sonography. Ultrasonography with phased array probe showed a large hyperechogenic lesion with a well-defined stalk that was originated from septum (the video is available). Brain computed tomography scan and chest X-ray did not show any abnormal findings. The patient was emergently transferred to a cardiac surgery center and underwent operation.

**Conclusions:** Atrial myxoma is the most common primary cardiac tumor that can lead to sudden death. Because of nonspecific symptoms such as weakness and syncope, early diagnosis may be difficult. Echocardiography is the method of choice for the diagnosis. This case showed the importance of emergency sonography in emergency patient management.

**Keywords:** Syncope, Bedside sonography, Myxoma

**Informed consent** This study was conducted in accordance with the ethical standards dictated by applicable law. Informed consent was obtained from each owner for enrolment in the study and the inclusion in this article of information that could potentially lead to their identification.

## A43 Work-related repetitive use injuries in ultrasound fellows

### Kristine Robinson^1^, Clara Kraft^1^, Benjamin Moser^1^, Stephen Davis^1^, Shelley Layman^1^, Yusef Sayeed^1,2^, Joseph Minardi^1^

#### ^1^Department of Emergency Medicine, West Virginia University School of Medicine, Morgantown, WV, USA; ^2^Department of Occupational Medicine, West Virginia University School of Medicine, Morgantown, WV, USA

##### **Correspondence:** Kristine Robinson, e-mail: kmagabo@hsc.wvu.edu

*Critical Ultrasound Journal* 2016, **8(Suppl 1)**:A43

**Purpose:** Extensive studies have reviewed work-related musculoskeletal (WRMSK) disorders among sonographers due to repetitive transducer manipulations and body positioning. To date, no study has investigated the prevalence of WRMSK complaints among Emergency Medicine Ultrasound (EMUS) Fellows.

**Methods:** A list of EMUS Fellowship programs was obtained from eusfellowships.com. An anonymous survey was sent to the fellows of 97 programs. The survey consisted of Likert scale responses regarding which WRMSK complaints occurred during training, program details, scanning habits, and demographic data. Data were collected using REDCap electronic data capture tools and analyzed using JMP Pro 11 software.

**Results:** Surveys were received from 67 out of 130 EMUS fellows (51 % response rate). Thirty-three fellows (53 %) were male. The average age was between 25–34 years (*n* = 48, 77 %). Fifty fellows (75 %) completed <7 scanning shifts a month; 65 (97 %) reported their shifts lasted <9 h. The average number of studies per shift varied widely, ranging from <12 to >30. Scanning duration typically lasted 5–10 min, with a rest cycle of 5–15 min. Most fellows disclosed standing and reaching over the patients to scan the other side, while appropriately adjusting patients and ultrasound machines. Fifty-eight (92 %) did not receive education on work-related or ergonomic injuries. Forty-five (67 %) experienced one or more WRMSK complaints (headache 18, eye 15, neck 25, upper-back 21, mid-back 21, lower-back 33, hip 8, knee 10, ankle/foot 10, shoulder 11, elbow 2, wrist 14, hand/finger 13), albeit most rated their problems as minor and did not affect their home life, work responsibilities, sleep, or psychosocial well-being. WRMSK complaints were usually noticed by 3 months. Twelve fellows reported preexisting complaints and were excluded from statistical analysis. Fellows with six or more scanning shifts per month were significantly more likely to report a WRMSK complaint (83 vs. 42 %; *p* = 0.0024, Fisher’s exact test). Fellows performing >20 studies per scanning shift were significantly more likely to report a WRMSK complaint (90 vs. 53 %, *p* = 0.0385).

**Conclusions:** EMUS fellows who perform more studies per shift or more scanning shifts per month are at a higher risk for WRMSK problems. EMUS Fellowship programs need to better educate their fellows on WRMSK disorders and ergonomic issues.

## A44 Lung ultrasonography in the evaluation of pneumonia in children

### Irmina Sefic Pasic^1^, Amra Dzananovic^1^, Anes Pasic^2^, Sandra Vegar Zubovic^1^

#### ^1^Radiology Clinic, Clinical Center University of Sarajevo, Sarajevo, Bosnia and Herzegovina; ^2^Oncology Clinic, Clinical Center University of Sarajevo, Sarajevo, Bosnia and Herzegovina

##### **Correspondence:** Irmina Sefic Pasic, e-mail: irmina.sefic@gmail.com

*Critical Ultrasound Journal* 2016, **8(Suppl 1)**:A44

**Purpose:** The aim of this paper is to introduce ultrasound features of community-acquired pneumonia found in pediatric population and to compare accuracy and specificity of lung ultrasound and X-ray.

**Methods:** Lung ultrasound was performed prospectively (February 2015 to May 2016) in 30 children (19 boys, 11 girls) with positive clinical and laboratory signs of impaired pulmonary function, suspected of pneumonia, and ultrasound findings were compared with chest X-ray findings. The mean age was 7.8 years. All ultrasound examinations were performed with high-frequency linear array probes of 7.5 and 10 MHz. The right and left lung were examined with anterior, posterior, and lateral approach, and each lung has been divided into 5 areas for precise localization of pathological condition. Interpretation of findings has been done by two radiologists, with one blinded to X-ray study and vice versa, and the results were compared using statistical analysis, so the specificity and sensitivity of the ultrasound were calculated.

**Results:** In the cohort of 30 patients (60 radiological findings), bacterial pneumonia was confirmed in 27 (90 %) patients. On ultrasound, we found pathological findings presented with vertically oriented ‘comet-tail’ artifacts in the lungs—B lines, consolidation of lung parenchyma with dynamic and static air bronchogram, which enables differentiation between atelectasis, pleural effusion, empyema, cystic formations in the lung parenchyma, and abnormalities of pleural line. Sensitivity of ultrasound was 100 %, specificity 75 %, positive predictive value 98 %, and negative predictive value of chest X-ray was 70.5 %. Relatively low specificity of lung ultrasound can be explained with the fact that consolidations of lung parenchyma found in two patients have been differentiated as tuberculous infections, so these findings in statistical analysis were considered as false positive.

**Conclusions:** Lung ultrasonography is a reliable tool for diagnosing pneumonia in children. It is suitable for routine use, diagnosis, and follow-up and may potentially decrease the total number of chest radiographs or it can eventually replace chest radiography in mild and uncomplicated cases of pneumonia in children.

## A45 Central venous catheter placement with the ultrasound aid: two years’ experience of the Interventional unit, Division of Intensive Care Medicine, KBC Zagreb

### Ana Godan Hauptman, Marijana Grgic Medic, Ivan Tomic, Ana Vujaklija Brajkovic, Jaksa Babel, Marina Peklic, Radovan Radonic

#### Division of Intensive Care Medicine, University Hospital Zagreb, Zagreb, Croatia

##### **Correspondence:** Ana Godan Hauptman, e-mail: ana.godan@gmail.com

*Critical Ultrasound Journal* 2016, **8(Suppl 1)**:A45

**Purpose:** To demonstrate 2 years’ experience of ultrasound-guided central venous catheterization team.

**Methods:** Central venous catheter (CVC) placements with the ultrasound aid in our interventional unit in 2 years (2014 and 2015) were analysed. Requests for CVC placement were mainly inquired after unsuccessful attempts in other departments or if the procedures were considered risky or difficult. Catheters placed in our ICU patients were not included. After ultrasound assessment of all potential sites, the optimal approach was chosen considering clinical data. The majority of CVC placements were done by young specialists.

**Results:** Jugular catheters were placed under direct ultrasound guidance by out-of-plane technique and transverse vein scanning. Subclavian catheters were usually placed by ultrasound-assisted cannulation. If the attempt failed, ultrasound-guided cannulation by in-plane technique and longitudinal vein scanning followed. There were 599 requests for CVC placement received. In 11 cases attempts were unsuccessful. The reasons were severe bleeding from the injection site in 4 cases, very narrow veins in 5 cases and suspected thrombosis in 2 cases. In 1 case, the procedure did not start due to patient’s agitation. Out of 587 placed CVCs, 358 subclavian, 217 jugular and 12 femoral routes were used. In 542 cases, CVCs were placed successfully at the first chosen site. In 45 cases, the site was changed after failure at the first attempted site. Reasons for changing the site were development of local hematoma in 12 cases, very narrow veins in 11 cases, very deep veins in 9 cases, inability to introduce the wire after successful vein puncture in 12 cases and inability to introduce catheter after successful placement of wire in 1 case. There were 2 pneumotorax cases without need for drainage and no other serious mechanical complications occurred. In 21 cases, the preferred site (subclavian) was not attempted but 20 jugular and one femoral sites were chosen.

**Conclusions:** By using ultrasound and careful education, we can report high success rate and low mechanical complication rates at CVC insertion even in selected potentially more challenging patients. Jugular approach was the most common second choice after initial subclavian attempt failure and has the highest success rate.

## A46 Duplicitas casui: two patients admitted due to acute liver failure

### Vedran Radonic^1^, Ivan Tomic^2^, Luka Bielen^2^, Marijana Grgic Medic^2^

#### ^1^Dom zdravlja Centar, Zagreb, Zagreb, Croatia; ^2^Division of Intensive Care Medicine, KBC Zagreb, Zagreb, Croatia

##### **Correspondence:** Vedran Radonic, e-mail: veve1x@gmail.com

*Critical Ultrasound Journal* 2016, **8(Suppl 1)**:A46

**Purpose:** To demonstrate the ability of interns to use ultrasound usefully.

**Methods:** Within 3 days, two patients were admitted to Intensive Care Unit (ICU) due to acute hepatic failure. In both, ultrasound examination performed by intern, who recently attended USLS-BL1, demonstrated dilated inferior vena cava (IVC) and hepatic veins and significant myocardium dilatation with reduced systolic function.

**Results:** Three months before admission, a 44-year-old male was hospitalized in another institution due to bilateral lung infiltrates. Mycobacterium bacillus was seen in sputum smear. Hence, antituberculotics were started. Bicuspid aortic valve and suspicious endocarditis were found on echocardiography. Ejection fraction was 40 %. In another institution endocarditis was excluded. Antituberculotics were continued but his clinical condition deteriorated with the development of acute liver failure. Lactate was 12.1 mmol/L, bilirubin 69 μmol/L, INR 2.79, AST 1648 U/L and ALT 1996 U/L, and he was transferred to our Department. Ultrasound demonstrated dilated IVC and hepatic veins as well as significant heart enlargement with reduced systolic function. Echocardiography showed severe aortic stenosis and EF of 20 %. After treatment of congestive heart failure and discontinuing antituberculotics, his liver function improved and a few weeks later, he underwent aortic valve replacement. A 54-year-old male was transferred to ICU from Gastroenterology ward due to overall clinical worsening. Recently, duloxetine was introduced for depression. Laboratory workup revealed high lactate 8.4 mmol/L, bilirubin 86 mmol/L, INR >6.0, AST 9302 U/L and ALT 6549 U/L. Transesophageal ECHO showed biventricular noncompactional cardiomyopathy with an EF of 20 %. After treatment of congestive heart failure (and stopping duloxetine), liver function improved. He was transferred to Cardiology ward.

**Conclusions:** Interns were stimulated to perform orientational ultrasound screening as an extension of the physical examination. In these two cases, liver congestion due to heart failure was recognized by orientational ultrasound as a significant contributing causative factor. Interns performing orientational ultrasound contributed to a proper understanding of the patients’ pathophysiology which had direct implications on patient management.

**Informed consent** This study was conducted in accordance with the ethical standards dictated by applicable law. Informed consent was obtained from each owner for enrolment in the study and the inclusion in this article of information that could potentially lead to their identification.

## A47 A pilot survey on an understanding of Bedside Point-of-Care Ultrasound (POCUS) among medical doctors in internal medicine: exposure, perceptions, interest, and barriers to training

### Peh Wee Ming

#### Singapore General Hospital, Singapore, Singapore

##### **Correspondence:** Peh Wee Ming, e-mail: pehwm86@gmail.com

*Critical Ultrasound Journal* 2016, **8(Suppl 1)**:A47

**Purpose:** Clinical bedside Point-of-Care Ultrasonography (POCUS) is an important adjunct to history and physical examination. In Singapore, there is no formal training or available literature assessing the current exposure, competency, and interest of internal medicine residents and internists to POCUS. The objective of this pilot survey is to assess the level of exposure, perceptions, interest levels, and possible barriers toward training of POCUS in internal medicine in Singapore.

**Methods:** In October 2015, all medical doctors who were working in the Singapore General Hospital Internal Medicine Department were invited to complete a hard copy printed 27-question Likert scale survey. Participation in the survey was voluntarily and the replies were kept confidential.

**Results:** A total of 124 medical doctors participated in the survey (response rate 82.1 %). The proportion of participants who have heard, witnessed, and performed POCUS were 65.6 % (*N* = 82), 71.2 % (*N* = 89), and 41.6 % (*N* = 52), respectively. POCUS was rated highly on usefulness in the practice of internal medicine (*M* = 8.74; SD = 1.34). The top 3 POCUS skills that doctors would like to acquire would be (1) procedural guidance POCUS(70.8 %), (2) point-of-care cardiac ultrasound(69 %), and (3) lung ultrasound (58.4 %) (based on  % ranked 1st–3rd). The sample mean of interest in undergoing further training in POCUS is 8.91 (SD = 1.27) (0 = not interested, 10 = very interested). The top 3 barriers identified were the (1) lack of an ultrasound machine (*M* = 7.98 SD = 2.28), (2) cost of the ultrasound machine (*M* = 7.79 SD = 2.19), and (3) lack of formal training curriculum (*M* = 7.25 SD = 2.08) (0 = not a barrier at all, 10 = severe barrier).

**Conclusions:** There is a high level of exposure and interest to pursue training in POCUS. Doctors perceived bedside POCUS as very useful in the practice of internal medicine. A lack of machine and training program impedes the development of a training program. This pilot survey may serve as a basic needs assessment to the implementation of an internal medicine POCUS training curriculum.

## A48 Unusual case of defecation syncope

### Nur hafiza Yezid, Fatahul Laham Mohammed

#### Hospital Sultanah Bahiyah, Alor Setar, Malaysia

##### **Correspondence:** Nur hafiza Yezid, e-mail: fizayezid@yahoo.com

*Critical Ultrasound Journal* 2016, **8(Suppl 1)**:A48

**Background:** Pulmonary embolism is a medical emergency that frequently underestimated, underdiagnosed, and undertreated disease. It an be presented with common symptoms that can be missed such as syncope. Untreated pulmonary embolism can have fatal consequences. Transthoracic sonography may serve as an additional method in the diagnostic workup of suspected pulmonary embolism.

**Case report:** We report a 74-year-old man with sudden dyspnea preceded by near-syncope at 6 a.m. being constrained. There were no other associated symptoms, no chest pain, and no fever. The patient was apparently well and ambulating well without support. On further history we found, that patient was discharged 3 days prior, from ward for post stroke seizure. No neurological deficit was present and he was ambulating well without support since discharge. His vital signs were BP 123/67, HR 120, and sPO_2_ 92 % on RA; however clinically he was not in respiratory distress. Patient was diagnosed by a senior medical officer as having acute coronary syndrome with fluid overload by evidence of bilateral ankle oedema and elevated JVP; however, the lung fields were clear and ECG was normal. Patient was also diagnosed by an emergency physician and bedside scan revealed dilated right heart, distended IVC, and dry lungs (A lines) bilaterally. The patient later underwent a CTPA which revealed extensive acute pulmonary embolism. Then he was treated with heparin infusion and subsequently discharged well with warfarin.

**Conclusion:** Pulmonary embolism is not a well-recognized cause of defecation syncope or defecation-associated sudden death. A large series of pulmonary embolism patients, up to 13 %, presented with syncope and less than 20 % of them had defecation syncope. Triple point-of-care ultrasound (lung, heart, and leg vein ultrasound) is a valuable alternative diagnostic tool in diagnosing pulmonary embolism in suspected pulmonary embolism and it may allow earlier diagnosis and treatment for pulmonary embolism.

**Informed consent** This study was conducted in accordance with the ethical standards dictated by applicable law. Informed consent was obtained from each owner for enrolment in the study and the inclusion in this article of information that could potentially lead to their identification.

## A49 A case report of massive pulmonary embolism; a multidisciplinary approach

### Zainal Abidin Huda, Wan Nasarudin Wan Ismail, W.Yus Haniff W.Isa, Hashairi Fauzi, Praveena Seeva, Mohd Zulfakar Mazlan

#### Anaesthesiology Department, University Science of Malaysia, Kota Bharu, Malaysia

##### **Correspondence:** Zainal Abidin Huda, email: hudaz82@gmail.com

*Critical Ultrasound Journal* 2016, **8(Suppl 1)**:A49

**Purpose:** The use of echocardiography as a rapid aid tool helps in deciding the direction of management of massive pulmonary embolism.

**Methods:** A patient was presented in the emergency department and an unknown aetiology of shock was screened for evidence of pulmonary embolism via bedside echocardiography.

**Results:** Based on the echocardiography results, the patient was successfully resuscitated and thrombolysed with Intravenous Tenecteplase, and regained her full level of consciousness after 11 days of ICU care.

**Conclusions:** In summary, we agreed that while bedside echocardiography may represent a potential useful screening technique for patients with PE with cardiogenic shock who require immediate intervention, nevertheless it is less applicable in an elective diagnostic strategy in haemodynamically stable, normotensive patients.

