# Erratum to: 12th WINFOCUS world congress on ultrasound in emergency and critical care

**DOI:** 10.1186/s13089-016-0050-z

**Published:** 2016-10-24

**Authors:** Edina Ćatić Ćuti, Nadan Rustemović, Dražen Perkov

**Affiliations:** 1 General Hospital Zabok and Hospital of Croatian Veterans, Zabok, Croatia; 2 University Hospital Zagreb, Zagreb, Croatia

## Erratum to: Crit Ultrasound J 2016, 8(Suppl 1):12 DOI 10.1186/s13089-016-0046-8

Following publication of the original article [1], it was brought to our attention that the third author, Dražen Perkov, was missing from the author list of A15 (*The challenge of AAA: unusual case of obstructive jaundice*). We would like to apologise for any inconvenience caused.
